# Intraglomerular crosstalk elaborately regulates podocyte injury and repair in diabetic patients: insights from a 3D multiscale modeling study

**DOI:** 10.18632/oncotarget.12233

**Published:** 2016-09-24

**Authors:** Hua Tan, Hualin Yi, Weiling Zhao, Jian-Xing Ma, Yuanyuan Zhang, Xiaobo Zhou

**Affiliations:** ^1^ Center for Bioinformatics and Systems Biology, Department of Radiology, Wake Forest School of Medicine, Winston-Salem, NC 27157, USA; ^2^ Key Laboratory of Gene Engineering of the Ministry of Education, State Key Laboratory of Biocontrol, School of Life Sciences, Sun Yat-sen University, Guangzhou, 510275, China; ^3^ Institute for Regenerative Medicine, Wake Forest School of Medicine, Winston-Salem, NC 27157, USA; ^4^ Department of Physiology, University of Oklahoma College of Medicine, Oklahoma, OK 73104, USA; ^5^ College of Computer Science and Software Engineering, Shenzhen University, Shenzhen, China

**Keywords:** diabetic nephropathy, glomerulus, podocyte regeneration, multi-scale model, multi-agent model

## Abstract

Podocytes are mainly involved in the regulation of glomerular filtration rate (GFR) under physiological condition. Podocyte depletion is a crucial pathological alteration in diabetic nephropathy (DN) and results in a broad spectrum of clinical syndromes such as protein urine and renal insufficiency. Recent studies indicate that depleted podocytes can be regenerated via differentiation of the parietal epithelial cells (PECs), which serve as the local progenitors of podocytes. However, the podocyte regeneration process is regulated by a complicated mechanism of cell-cell interactions and cytokine stimulations, which has been studied in a piecemeal manner rather than systematically. To address this gap, we developed a high-resolution multi-scale multi-agent mathematical model in 3D, mimicking the *in situ* glomerulus anatomical structure and micro-environment, to simulate the podocyte regeneration process under various cytokine perturbations in healthy and diabetic conditions. Our model showed that, treatment with pigment epithelium derived factor (PEDF) or insulin-like growth factor-1 (IGF-1) alone merely ameliorated the glomerulus injury, while co-treatment with both cytokines replenished the damaged podocyte population gradually. In addition, our model suggested that continuous administration of PEDF instead of a bolus injection sustained the regeneration process of podocytes. Part of the results has been validated by our *in vivo* experiments. These results indicated that amelioration of the glomerular stress by PEDF and promotion of PEC differentiation by IGF-1 are equivalently critical for podocyte regeneration. Our 3D multi-scale model represents a powerful tool for understanding the signaling regulation and guiding the design of cytokine therapies in promoting podocyte regeneration.

## INTRODUCTION

The chronic kidney disease (CKD), resulting in end stage renal disease (ESRD), has become a significant health problem worldwide. According to the 2014 USRDS annual report [1], an estimated 13.6% of adults in the United States have CKD, costing $44.6 billion of medical care expenditure and taking up 19.6 percent of all Medicare parts A and B spending. Diabetic nephropathy (DN) occurs in up to 40% of patients with diabetes. It is estimated to affect about seven million people in the United States [2]. Adults with diabetes have a higher risk of developing DN than those without this disease. Although the time of clinical presence of DN varies between type 1 and 2 diabetics, pathophysiological progresses in both conditions are similar [3].

It has been recently established that most renal pathologies that ultimately result in ESRD originate within the glomerulus. The glomerulus comprises a network of capillaries located at the beginning of a nephron in the kidney [4]. The nephron is the basic structural and functional unit of the kidney which plays a critical role in eliminating wastes from the body, regulating blood volume/pressure, and controlling levels of electrolytes and metabolites in blood. Since the glomerulus serves at the first stage in the filtering process of blood carried out by the nephron, its normal function is essential for the healthy status of the whole kidney.

**Figure 1:**
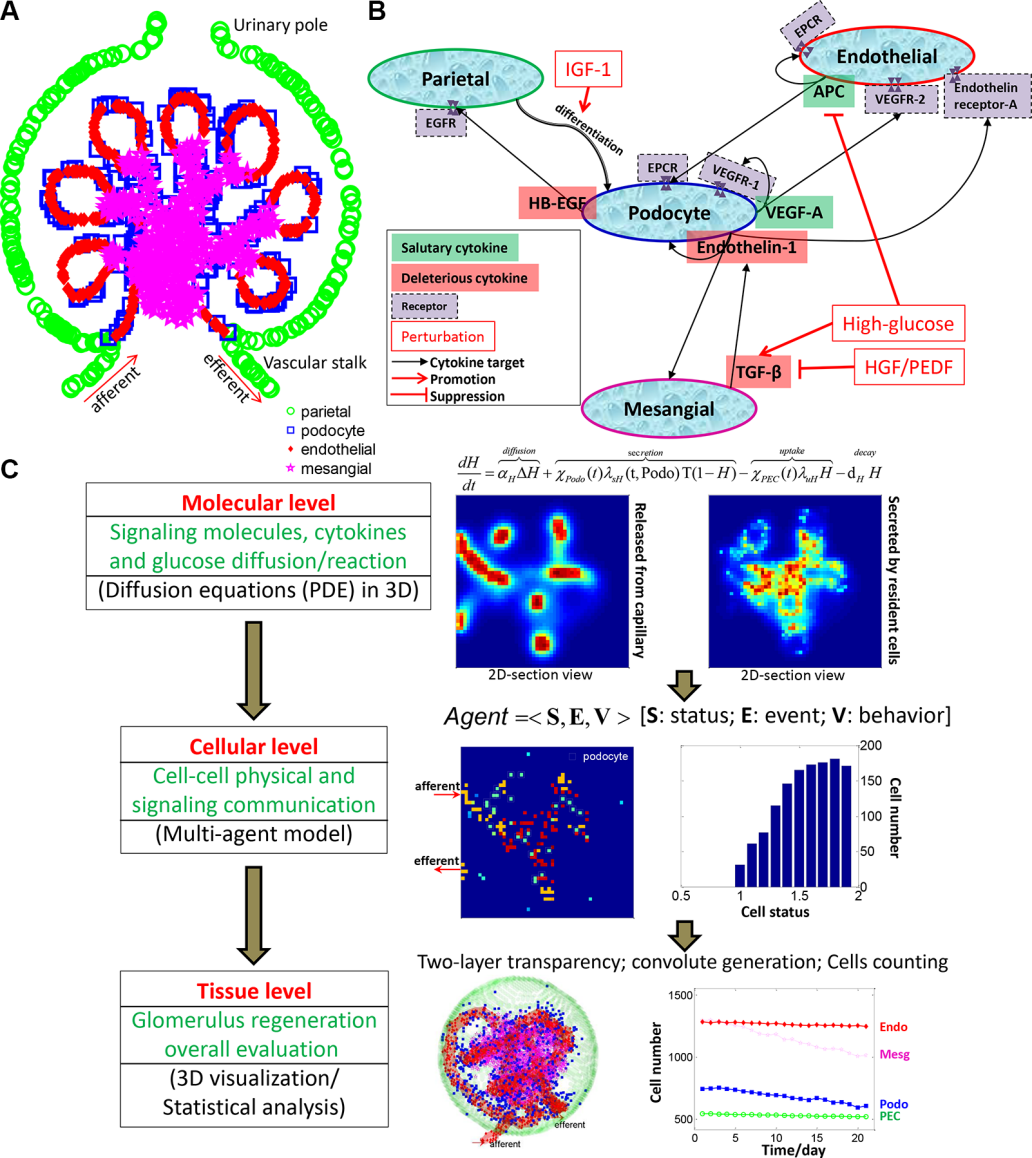
Overview of the 3D multi-scale multi-agent systems model for podocyte regeneration (**A**) Schematic depicting the anatomical structure and cellular composition and distribution of the glomerulus. (**B**) Major intercellular signaling communications between glomerular cells. (**C**) Overall framework and specific implementation procedures of the proposed multiscale (molecular-cellular-tissue) systems model.

The glomerulus has a complicated structure with two major compartments, the glomerular capillary tuft and the so-called Bowman's capsule which surrounds the capillary tuft. Four major types of cells reside in the glomerulus. As illustrated in Figure 1A, podocytes, endothelial cells and mesangial cells are residing in the glomerular tuft, while the parietal epithelial cells (PECs) compose the outer layer of the capsule [5]. Glomerular cells communicate with each other to maintain normal kidney function through particular signaling molecules, and respond to various injuries such as hyperglycemia in DN conditions [5–7] (Figure 1B).

Converging evidence has indicated that depletion of podocytes is a common determinant that leads to a broad spectrum of clinical syndromes in the kidney [4, 8]. Many reasons can lead to loss of podocytes, including toxic, genetic, immune, infectious, oxidant, metabolic and hemodynamic factors [9]. As long as the podocyte loss is limited, restitution or repair is possible. Once the loss of the glomerular podocytes exceeds a certain threshold (approximately 30%), glomerulosclerosis ensues [10].

Recently, multiple studies have consistently corroborated that the depleted podocytes can be regenerated via differentiation of the adjacent PECs [4, 5, 8, 11, 12]. Hence, the PECs serve as the local progenitors of podocytes and reconstitute the podocyte population to a normal level following podocyte loss. Since the cells within the glomerular tuft have an influence on each other via a multitude of paracrine mechanisms [7], podocyte injury and repair are also regulated by the complex mechanism of cell-cell interactions and cytokine stimulations. Several prominent pathways of para- and auto-crine crosstalk between glomerular cells in response to damage have been demonstrated [6]. And disruption of any of these pathways leads to podocyte apoptosis, which constitutes a driving force for progression of various renal diseases [6].

Experimental efforts have previously been devoted to exploring the signaling regulations between different glomerular cells. Particularly, vascular endothelial growth factor A (VEGF-A) produced by podocytes is essential for maintenance of actin cytoskeleton and endothelial integrity [13, 14]. Heparin-binding epidermal growth factor-like growth factor (HB-EGF) is secreted by injured podocytes to promote PEC proliferation [15]. Hyperglycemia-induced TGF-β elicits endotheilin-1 formation in podocytes, resulting in endothelial dysfunction and mesangial expansion, as well as podocyte injury [16]. Activated protein C (APC) produced by endothelial cells protects themselves and podocytes from apoptosis [17]. These major signaling communications between glomerular cells are shown in Figure 1B. Since most of the previous studies focused on cell-cell interactions and cytokine regulations in a piecemeal manner, the whole process of podocyte regeneration has not yet been well elucidated at a systems level.

In this work, we systematically investigated the intercellular communications within the glomerulus and explored how these cell-cell interactions contribute to the regulation of podocyte injury and repair. We also tested the influence of external cytokine perturbations (singly or in combination) on the regeneration process in diabetic nephropathy (DN). We constructed a high-resolution multi-scale and multi-agent model in 3D to mimic the real glomerulus micro-environment. As shown in Figure 1C, we employed a system of partial differential equations (PDEs) to depict the production, diffusion, and degradation processes of each important signaling molecule at the molecular scale. The glomerular cells secrete and/or bind to particular signaling proteins at dynamic rates depending on their health status. Therefore, the molecular system is closely associated with the cellular scale. At the cellular scale, we developed a novel agent-based model to simulate the behaviors of individual glomerular cells with or without cytokine perturbations. These external perturbations activate related downstream signaling cascades, which further influence cell fate decisions. The molecular and cellular profiles together determine the dynamic change at the tissue level. The constructed 3D micro-environment paved the way for subsequent visualization and statistical analysis of the podocyte regeneration process.

This systems model was constructed by integrating scattered information obtained through a wealth of previous individual studies to generate a consolidated system, enabling a comprehensive investigation of the intra-glomerular crosstalk at the systems level. Following calibration of the parameters with specific experimental data at particular biological scales, our model recapitulated critical experimental observations, and further led to insightful new discoveries. Some of our findings have been verified by independent *in vivo* experiments, while others can be validated in the clinic. Overall, our proposed 3D multi-scale model provides a powerful tool for integrating existing biological knowledge and experimental observations for new discoveries with respect to molecular mechanisms of kidney disease. Therefore, our model is beneficial for generating testable hypotheses and designing cytokine perturbations to improve podocyte regeneration in clinical practices.

## RESULTS

### Cell dynamics in a 3D glomerulus under physiologic and diabetic conditions

**Figure 2:**
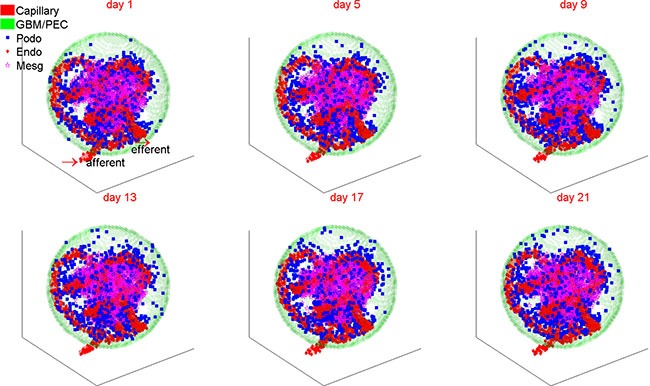
A representative simulation of 3D glomerular cell dynamics under normal condition The number of podocytes is dynamically balanced owing to continuous replenishment from the resident parietal epithelial cells under normal physiological conditions. PEC: parietal epithelial cell; Podo: podocyte; Endo: endothelial cell; Mesg: mesangial cell.

To study the podocyte injury and regeneration processes upon exogenous cytokine perturbations, we need first to calibrate a simulation platform capable of recapitulating the cellular dynamics under physiologic (normal) and diseased conditions without any treatment. In addition, this simulation system should also be able to reproduce the molecular changes following glomerulus dysfunction as observed in experimental studies and in clinic. Figure 2 illustrates a typical simulation result under physiologic conditions for a period of 3 weeks. Although podocytes were lost due to natural detachment, the parietal epithelial cells, located at the glomerular basement membrane (GBM, green), replenished the podocyte loss by differentiation and maintained the balance of podocyte cell numbers under normal physiological condition. As shown in Figure 2, PECs (green) differentiated into podocytes (blue) and migrated from the capsule membrane to the podocyte region surrounding the capillary tuft. The endothelial (red) and mesangial (magenta) cells could also migrate, but were largely confined to the regions they reside in (Materials and Methods). In addition, all types of cells were upheld at healthy status under physiologic condition (Supplementary Figure S1), in line with typical dynamic equilibrium of essential signaling proteins (cytokines) observed in reality: concentration of salutary cytokines (VEGF-A and APC) maintained at high concentration, while deleterious proteins (HB-EGF, endothelin-1 and TGFβ) were constantly suppressed under physiologic conditions (Supplementary Figure S2).

**Figure 3:**
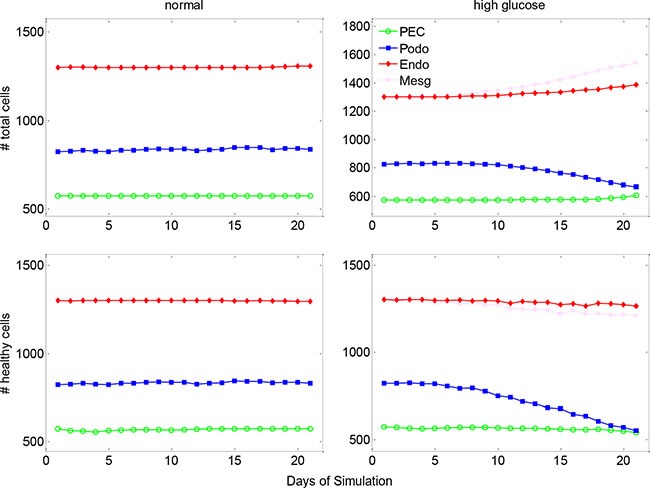
The dynamic changes of glomerular cell numbers under normal and high glucose conditions The top row shows the dynamic change of total glomerular cells and the lower row for healthy cells under normal (left column) and high glucose (right column) conditions. PEC: parietal epithelial cell.

Figure 3 shows the changes in cell number over 21 days of simulation under physiologic and diabetic (high glucose) conditions. In the diabetic scenario, simulated high concentration of glucose was diffused from the micro-vasculatures to the glomerulus (Supplementary Figure S3). Contrary to the well-balanced numbers of glomerular cells under physiologic condition, the numbers and health status for all four types of glomerular cells became abnormal under hyperglycemic condition. The healthy cells of all four types were diminished to various extents, which can be further discerned from Supplementary Figure S4. The total (including healthy and unhealthy) PECs, endothelial and mesangial cells were substantially increased by 6%–19% under high glucose condition, while the total podocytes were depleted by 20% compared to the normal condition. These simulation results were well concordant with clinical observations referring to mesangial expansion and podocyte loss in type 1 diabetic patients [18].

**Figure 4:**
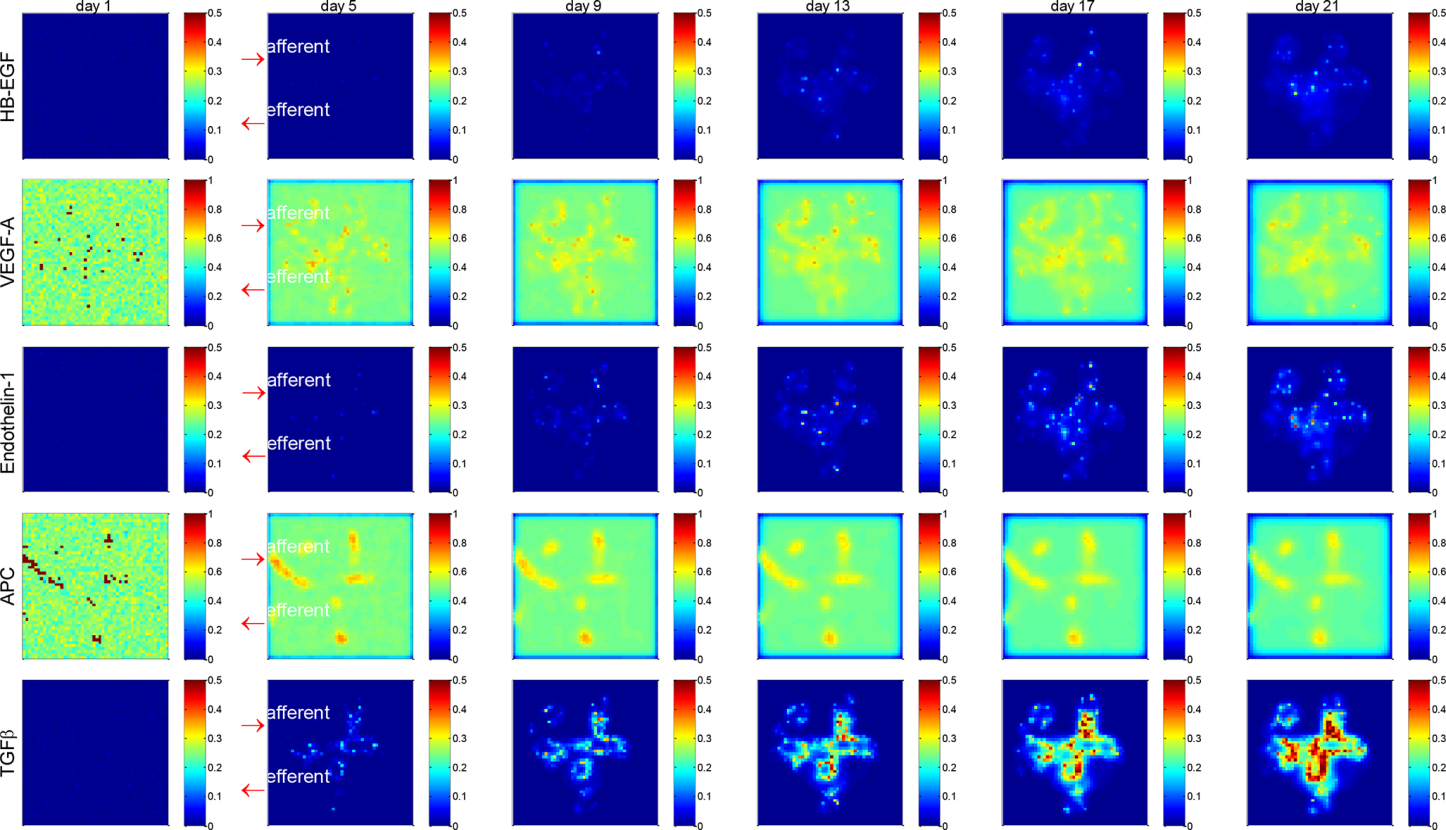
Spatial and temporal distribution of critical cytokines in a glomerulus under high glucose condition Shown is 2D section view of spatial distribution at y = 0. Deleterious cytokines HB-EGF, Endothelin-1 and TGFβ were accumulated and salutary signaling proteins VEGF-A and APC were gradually diminished over time. Red arrows indicate the afferent and efferent arterioles of the glomerulus. See Supplementary Figures S2, S6, S8, S10 for more results of spatial and temporal distribution of critical cytokines.

Corresponding to the cellular abnormality in cell number and cell health status, the signaling molecules and cytokines were actively altered in the glomerulus under diabetes-induced hyperglycemic condition. Figure 4 shows (in 2D section view) the substantial accumulation of the deleterious cytokines HB-EGF, endothelin-1 and TGFβ, and gradual attenuation of the salutary cytokines VEGF-A and APC within the glomerulus over disease progression. A quantitative representation of the molecular concentrations over simulation period is shown in Supplementary Figure S5. It appears that TGFβ concentration was significantly increased in the central area of the glomerulus, indicating enhanced extracellular matrix deposition and mesangial expansion during disease progression. These simulation results are comparable to previously reported experimental outcomes [19, 20]. Collectively, our simulation results at both cellular (Figure 3) and molecular (Figure 4) levels (no treatment) were largely corroborated by clinical or experimental observations. Therefore, the systems model establishes a well-calibrated simulation platform for the subsequent treatment perturbation studies.

### Podocyte regeneration under various cytokine treatments

**Figure 5:**
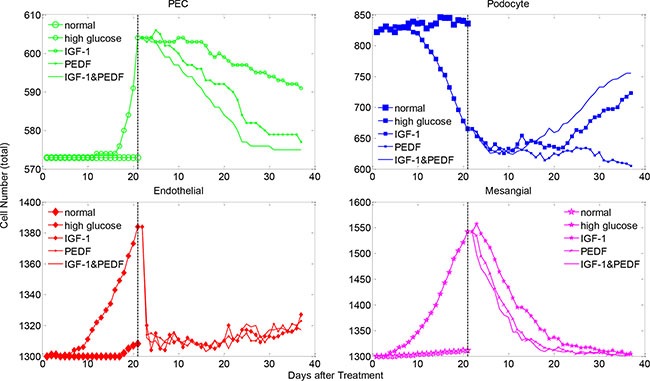
The changes in the total cell numbers under single and dual cytokine treatments Shown are total cell numbers of each glomerular cell type under denoted treatments after high-glucose induced injury. The cell number dynamics under normal and high glucose conditions are plotted for comparison. See Supplementary Figure S12 for the changes in the healthy cell numbers under various cytokine treatments.

We conducted a series of simulations to explore the effect of various regimes of cytokine treatment on the glomerulus restoration from diabetic injury. We started from an injured glomerulus system manifesting abnormal glomerular cells and aberrant concentrations of critical signaling molecules (described in above section), and simulated an external cytokine delivery into the glomerulus through the afferent arteriole. We assessed podocyte regeneration by monitoring the numbers of total and healthy cells over the simulation period. Figure 5 shows a representative simulation result of total cell number for three cytokine treatment scenarios, including (1) IGF-1 alone, (2) PEDF alone and (3) IGF-1 and PEDF combination treatment. A detailed illustration of the molecular and cellular dynamics under these treatments is shown in Supplementary Figures S6–S11. We repeated five times for each treatment scenario and explored the extent of output variation by uncertainty analysis (shown in below section). Generally, all three treatments restored the cell numbers from abnormal to normal level, i.e., total cell numbers of PECs, endothelial and mesangial cells were consistently decreased, while total podocytes were significantly increased following each treatment. It should be noted that PECs, endothelial and mesangial cells were expanded while podocytes were reduced during the hyperglycemic damage, explaining the opposite direction of recovery for different cells. We also assessed the dynamics of healthy cells. Although oscillation existed, the overall trend for the numbers of healthy cells is increasing, indicating a gradual recovery from injury (Supplementary Figure S12). These simulation results supported the assumption that PEDF and IGF-1 played dissimilar roles in restoring the glomerulus function from injury. Specifically, PEDF displayed higher potency in mediating the non-podocyte glomerular cells to a normal state, while IGF-1 proved more critical for promoting the differentiation of PECs into podocytes.

Taken together, combined treatment with two cytokines were more effective in promoting podocyte regeneration. The number of total and healthy podocytes was increased rapidly and dramatically in the group treated with IGF-1&PEDF, compared with the groups treated with IGF-1 or PEDF only (Figure 5 and Supplementary Figure S12). For the healthy status of other glomerular cells, the combined treatment scenario also displayed generally higher recovery rate than single cytokine treatment (Supplementary Figure S12). In addition, combined treatment with two cytokines exhibited a greater potency in restoring the total cell numbers of PECs and mesangial cells to a normal level by rapidly reducing their total numbers (Figure 5, green and magenta). However, no significant difference in total endothelial cells between treatment scenarios was observed (Figure 5, red). Interestingly, while the total PECs and mesangial cells were gradually reduced under various treatment conditions, the total number of endothelial cells experienced a sharp decrease followed by an obvious increase in the later stage of treatments. In addition, the trend of endothelial cell dynamics was in good agreement with a clinical outcome under similar treatment schedule, which will be detailed in the following section.

**Figure 6:**
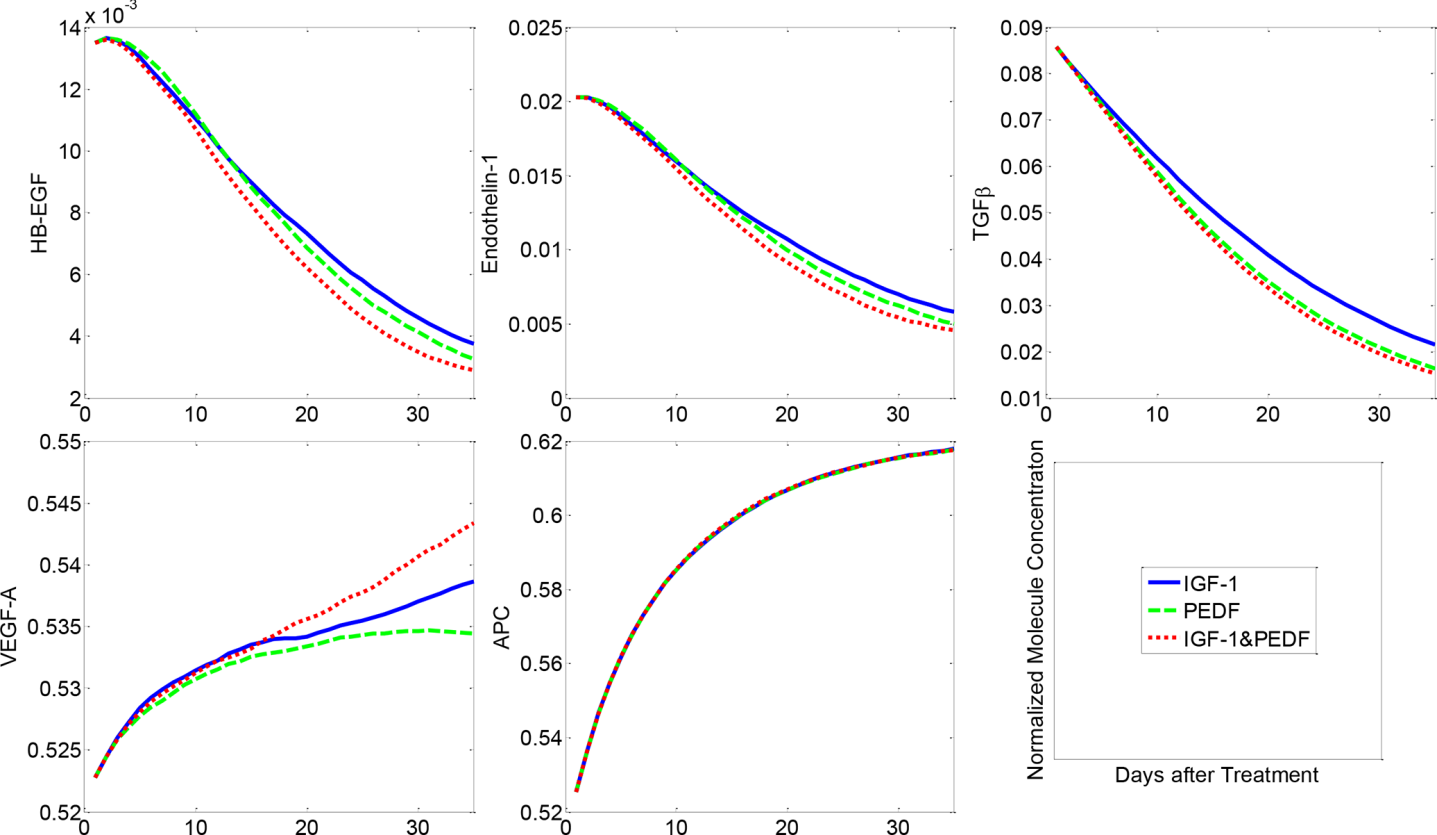
Molecular concentration dynamics under various cytokine treatments Combined treatment with two cytokines resulted in the most significant suppression of HB-EGF, Endothelin-1 and TGFβ, followed by the single treatment with PEDF or IGF-1. VEGF-A and APC were activated to various extents by different treatment regimens.

At the molecular level, the combined treatment with IGF-1 and PEDF significantly attenuated the levels of HB-EGF, endothelin-1 and TGFβ, compared to the treatment scenarios with IGF-1 or PEDF alone (Figure 6). When delivered alone, PEDF (dashed green) turned out to be more efficient in suppressing the levels of detrimental proteins (HB-EGF, Endothelin-1, TGFβ) than IGF-1 did (solid blue). Combined treatment (dotted red) showed the most potent suppression effect on these proteins. On the other hand, all three treatment regimens promoted the secretion of VEGF-A by healthy podocytes and APC by the healthy endothelial cells. The IGF-1 manifested greater potency in enhancing VEGF-A production than PEDF did. The production of APC was largely augmented in all of treatment groups to a similar extent. Overall, these results demonstrated the change of molecular profiles underlying the cellular dynamics in various treatment conditions.

### Treatment perturbations in comparison with experimental data

**Figure 7:**
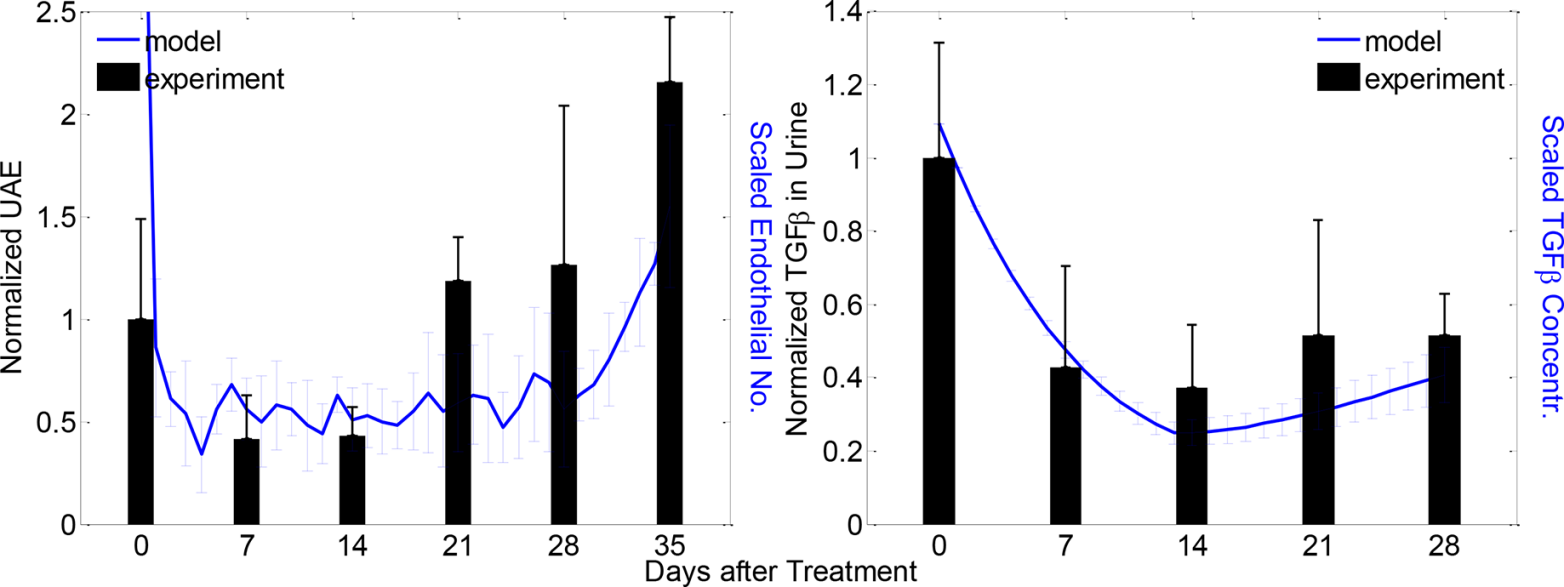
Modeled and experimental results of clinical outcome and molecular dynamics under a particular PEDF treatment scenario Left: dynamics of experimental UAE (black bars) and predicted endothelial cell numbers (blue curves) after the PEDF treatment; right: dynamics of experimental (black bars) and predicted (blue curves) TGFβ concentrations after PEDF treatment. Experimental and modeled cells/proteins were measured weekly and daily respectively for denoted periods (mean ± SD, *n* = 5 for both model and experiment results).

To validate our model, we conducted an alternative treatment schedule, which was comparable to our experimental condition. Specifically, we calibrated a PEDF treatment profile analogous to our previously published *in vivo* study [21]. In this treatment regime, PEDF concentration in the urine of diabetic rats was increased during the first two weeks after PEDF treatment, and then started to decline (Supplementary Figure S13). Since PEDF was delivered by adenovirus-mediated constructs in the *in vivo* experiments, the decrease of PEDF concentration in a later stage of treatment was assumedly due to the diminished virus particles. Under this treatment scenario, we found that the number of total endothelial cells was substantially decreased in the first two weeks after treatment, followed by a discernable increase during the next three weeks. Strikingly, the dynamic changes in the endothelial cell numbers were well consistent with the dynamics of urinary albumin excretion (UAE) measured in the diabetic rats (Figure 7 left). In addition, our simulation results at the molecular level were also consistent with the experiment results [21]. Specifically, the concentration of TGFβ was dramatically reduced in the following few weeks after treatment and then went back slightly (Figure 7 right). It should be noted that the experimental results of TGFβ showed greater variance at most time points in comparison with our simulation results, which was presumably attributable to the fact that the level of experimental TGFβ was determined from the urine sample instead of direct measurement of the tissue. The sampling timing and degree of injury could also contribute to the significant variance. The dynamics of endothelial cell number in our simulation was not strictly consistent with the UAE data from the *in vivo* experiments, but exhibited similar trend. This observation implied that the endothelial cell expansion was an important factor but not the sole determinant of high UAE measured in the diabetic rats.

### Sensitivity and uncertainty analysis of the systems model

**Figure 8:**
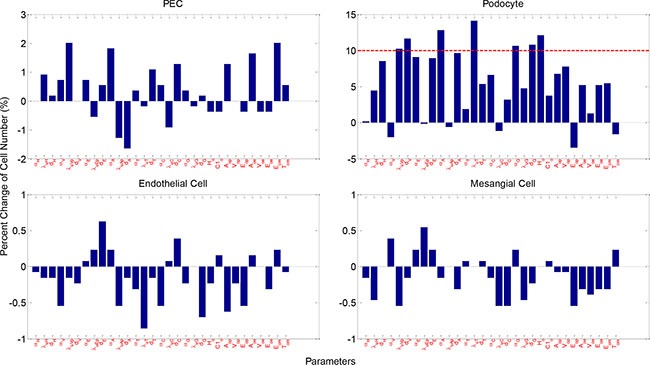
Parameter sensitivity analysis Shown are percent cell number change of healthy glomerular cells upon 10% increase in parameters. Physical meaning of the parameters are described in Supplementary Tables S1 and S2. See Supplementary Figures S14–S16 for more sensitivity analysis results.

To determine the sensitivity of the model output to the involved parameters, we conducted a thorough sensitivity analysis on the critical parameters (Materials and Methods). Figure 8 shows the changes in the number of healthy cells at week 5 upon a 10% increase of 31 critical parameters (21 for molecular PDEs and 10 for cellular agent-based module respectively) under IGF-1 and PEDF combined treatment. Less than 3% variation in the number of healthy PECs, endothelial cells and mesangial cells were observed, demonstrating a low sensitivity of these three cell types to the perturbed parameters. On the other hand, the change in the number of healthy podocytes were sensitive to APC diffusion rate (*α_A_*) and uptake rate of TGFβ by podocytes (*λ_uT_*), since 10% increase in either APC diffusion rate or TGFβ uptake rate led to ~13% increase in podocyte numbers. The number of podocytes were also marginally sensitive to the uptake rate of VEGF-A by podocytes (*λ_uVp_*), and the diffusion and degradation rate of glucose (*α_G_*,*d_G_*). Interestingly, the number of healthy podocytes was considerably increased with increased degradation rate of VEGF-A (*d_V_*). It was expected that an increase in the threshold of HB-EGF for PEC damage (*H_θ_*) would lead to an increase in the podocytes. However, the percent changes in the total numbers of podocytes appeared much less sensitive to the parameters perturbed. Only the perturbation on parameter *λ_uT_* yielded a 10% variation in the number of podocytes. The effect of parameter increase on total cell number, and the effect of parameter decrease on total and healthy number are displayed in Supplementary Figures S14–S16, respectively. Collectively, the parameter sensitivity analysis played an important role in identifying the critical factors that have significant impacts on the outcomes under particular treatment scenarios.

**Figure 9:**
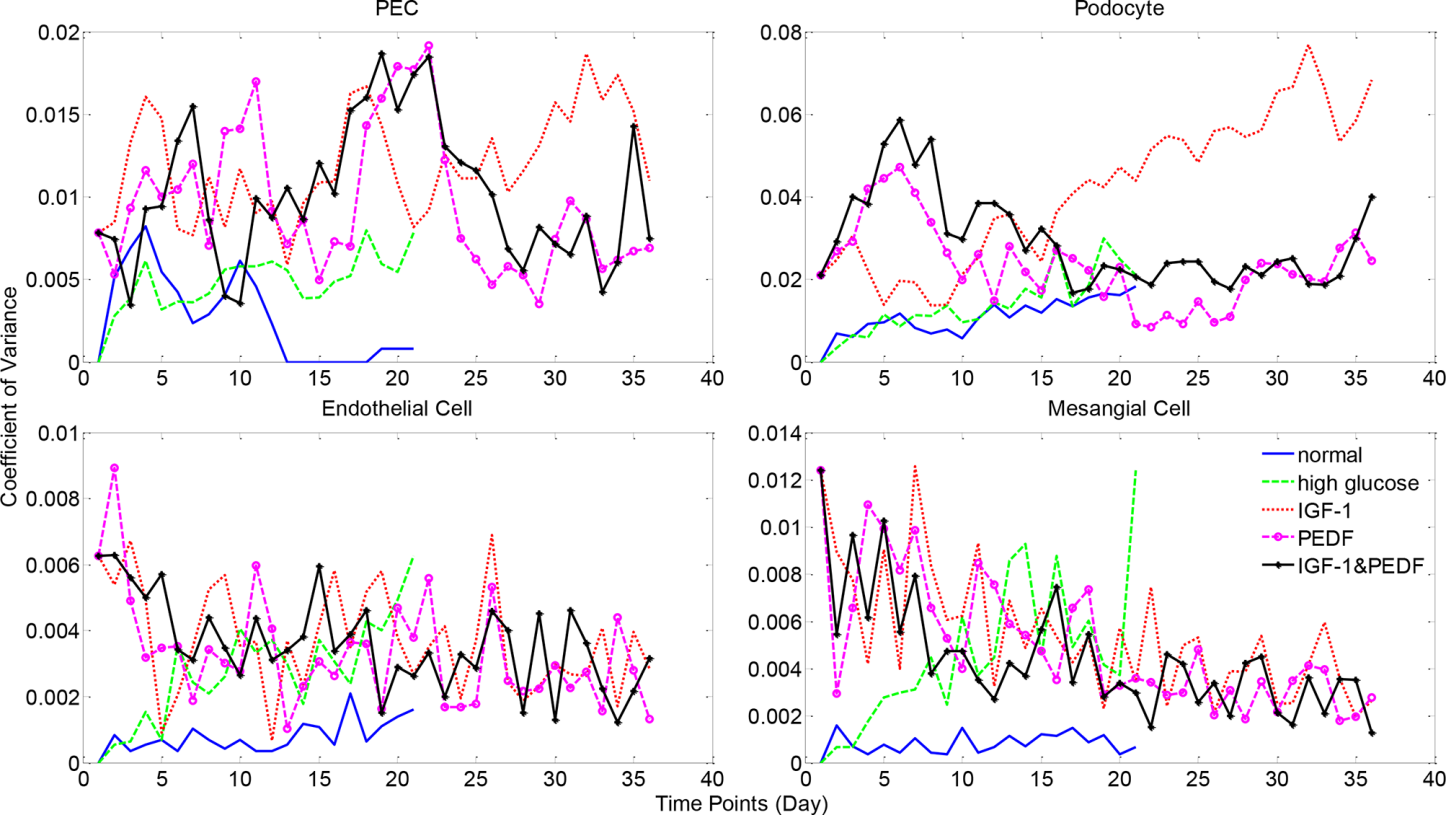
Model uncertainty analysis results for healthy cell numbers Simulations were repeated five times with identical parameter settings. Coefficient of variance (CV) was calculated as the ratio of standard deviation to mean cell numbers at each time point. See Supplementary Figure S17 for more uncertainty analysis results.

To further explore the model uncertainty potentially caused by the stochastic processes, we conducted uncertainty analysis via repeating the simulations for five times with identical parameter settings (Materials and Methods). Figure 9 illustrates the coefficient of variation (CV) over simulation time under various treatments, including the normal and high glucose conditions. The numbers of healthy glomerular cells displayed negligible variations under all simulation circumstances, with CV values invariably bounded by 0.1. The total number of PECs displayed certain variance (up to 0.25), but not significant (CV < 1 for all simulations, Supplementary Figure S17). These results indicated that the proposed model was quite stable despite the inherent randomness in the model.

## DISCUSSION

The renal glomerulus has an intricate structure and dysfunction will occur regardless of which components are affected by diseases [22]. The dysfunction in one cell type may gradually propagate to the other, due to cell-cell interactions (via paracrine mechanisms) within the glomerulus [6, 23, 24]. Previous studies have investigated the intercellular communications piece by piece and achieved piecemeal results for specific interests. The glomerulus is the basic functional unit of the kidney, and all glomerular cells orchestrate to play synergistic roles in the filtration of blood. Therefore, systematically modeling the dynamic interactions of all glomerular components can help us better understand their impacts on podocyte injury and repair. To our best knowledge, the proposed work represents the first attempt to integrate the current experimental results and clinical observations into a consolidated simulation and analysis system to facilitate exploration of the glomerulus injury and repair activities from an overall perspective.

Our simulation results highlighted the significance of highly balanced signaling transductions among glomerular cells in maintaining the normal function of the glomerulus. Under normal physiological conditions, the salutary cytokines (e.g. VEGF-A and APC) are maintained at a high level, while the levels of detrimental proteins, including HB-EGF, endothelin-1 and especially TGFβ, are effectively suppressed. Once damaged by disease conditions, such as diabetes, the resultant hyperglycemic condition promptly induces the production of TGFβ in mesangial cells, which in turn promotes endothelin-1 formation in podocytes. Endothelin-1 is a key factor that causes dysfunction of endothelial and mesangial cells, as well as podocytes [16]. The dysfunctional endothelial cells tended to secrete aberrantly less APC than normal, which will exacerbate the injury of podocytes. On the other hand, the damaged podocytes secreted HB-EGF, which in turn stimulates the activation and proliferation of PECs, and disturbs their compensatory differentiation towards podocytes. In summary, the intercellular crosstalk between glomerular cells regulates the healthy physiology of glomeruli in a very elaborate manner. Hence, interference of any node of the signaling network is likely to cause a chain reaction that may eventually lead to ill-conditioning of the glomeruli, including podocyte depletion, endothelial dysfunction and mesangial expansion, ECM accumulation and eventually glomerular hypertrophy.

The present study further suggested that amelioration of glomerulus stress and promotion of PEC differentiation are equivalently important for podocyte regeneration. Previous *in vivo* and *in vitro* studies have shown a beneficial effect of PEDF in preventing fibrosis, inflammation and angiogenesis processes in glomerulus, via inhibition of the expression of TGFβ, CTGF, and ECM proteins [21, 25, 26]. Hepatocyte growth factor (HGF) is another cytokine which plays a very similar role to PEDF in maintaining the glomerulus normal function [19, 27, 28]. We chose PEDF as a prototype cytokine in our study since the experimental data were available in multiple time points up to 5 weeks. Besides the cytokines-based therapeutics, another significant strategy for renal protection was the stem cell based therapy [29, 30]. Studies indicated that multipotent mesenchymal stem cells (MSC) improved renal function or renal cell survival *in vivo* and *in vitro* [31, 32]. In addition, MSCs exerted beneficial effects on renal cell repair via complex paracrine actions instead of differentiation into target cells directly. Particularly, the IGF-1 plays a vital role in sustaining stem cell mediated renal repair [32]. Our experimental results confirmed that PECs could be directed to podocytes by IGF-1 overexpression, confirmed by several podocyte-specific biomarkers (e.g. nepherin, podocin, synaptopodin and WT1 [33]) (data not shown). Although experimental data with combined IGF-1 and PEDF treatment is currently lacking, our proposed computational model, calibrated by clinical observations under a single cytokine treatment, displayed significant capability in prediction of the outcomes of the combined cytokine therapy (Figure 5).

We also tested an alternative model which integrates the convection effect of flow for the molecular diffusion model. By varying the convection coefficient by various orders of magnitude, the major model output under physiologic and hyperglycemic conditions did not change much comparing to the baseline model (supplementary materials and Supplementary Figures S19–S24). These results further confirmed our expectation that the convection term does not significantly impact the instant and relative distribution of the cytokines within the glomerulus, and hence rarely influence the dynamic change of cell numbers. However, the alternative model can also be applied in particular applications, especially when adequate data are available for model calibration. Otherwise, the alternative model can be adaptive to the baseline one by vanishing the convection coefficient, as shown in supplementary equations (1a-5a).

Mathematical modeling is increasingly becoming a valuable approach to understanding biological processes and allows us to pose and test hypotheses more efficiently than by wet-lab experiments alone [34, 35]. Our proposed systems model allows biophysical and pathophysiological properties to be adjusted quantitatively and incrementally, based on assumptions or clinical observations. In addition, our model enables subject-to-subject variability to be circumvented since identical or similar starting configurations could be set for a series of *in silico* experiments. It provides a better interpretation of biological experiments with the capability to trace the causal sequences in details. Therefore, our developed computational model is powerful for interpretation of the underlying molecular and cellular mechanisms of biological processes with clinical syndromes, such as the podocyte regeneration addressed in this study.

Multiscale modeling represents a promising strategy for exploring the system behavior mediated by components of various biological and/or functional scales [36]. In the past years, multiscale models have been widely and successfully applied in studying complex biological processes including tumor growth and tissue regeneration [37–39]. On the other hand, agent-based models have exhibited advantages in addressing complex systems with autonomous components [40]. Our systems model takes advantage of the merits from both modeling schemes for integrative simulation and analysis of the glomerular system. We adopted a hybrid modeling technique [41], referring to the continuous differential equations for the molecular scale and a discrete agent-base model for the cellular scale, to achieve this integration. Our model has satisfactorily addressed the coupling of the three biological scales (molecular, cellular and tissue scales) by incorporating the cross-scale interaction into the systems model. For instance, the production and diffusion of cytokines were affected by the number and healthy status of glomerular cells. We constructed a time-dependent characteristic function to describe cell status and incorporated it into the differential equations (PDEs) as a coefficient. Likewise, the impact of particular cytokines in the surrounding microenvironment on cell activities and fate decisions were implemented in the multi-agent module (Figure 1).

Genetic alteration is another important regulator of podocyte injury and repair. Greka and Mundel summarized a list of genes (e.g. NPHS1/2, SYNPO, WT1, etc.) which, if mutated, will affect the podocyte structure and function [9]. We also devoted intensive efforts to study the effect of gene mutation on the related protein function and the relevance of mutations to human diseases including cancer [42–44]. We will incorporate the genetic causes into the podocyte regeneration model, evaluate the quantitative correlation between genetic aberration and disease progression in the future studies.

Although we are the first to model the podocyte regeneration process under the context of the whole glomerulus, there has been plenty of work focusing on other components of the nephron [45]. Particularly, the dynamics of molecule (e.g. ion and water) transport and reabsorption within each segment of the nephron tubule have been studied individually by a series of ODE models [46–52]. The behavior of single-nephron and multiple-nephron systems was simulated by discrete network models with fluid transport laws [53–55]. Researchers also *in silico* reconstructed the 3D structures of nephron tubules and surrounding vessels for better understanding the role of 3D interactions between tubules and vessels in nephron biological processes [56, 57]. Other groups investigated the 3D construction of the glomerular capillary network and modeled the blood flow and filtration in the reconstructed glomerular capillary network [58, 59]. The existing models have been beneficial for studying the working principles of the nephrons, particularly the biophysical properties of ion and water transport, excretion and reabsorption at the microscopic level. However, the existing models seldom took account of the pathology of kidney diseases. In particular, the behaviors (including the intracellular and intercellular signaling regulations) of the nephron cells (especially the glomerular cells) were rarely explored using mathematical models. Our work addressed this gap by incorporating the signaling networks that connect all major glomerular cells into an integrative system, and brought new insights into the molecular mechanisms of podocyte regeneration.

## MATERIALS AND METHODS

### Molecular scale of the model: molecule diffusion within 3D glomerulus

In our systems model, the glomerular cells communicate with each other through secreting and binding particular cytokines that diffuse within the glomerulus. We depict the signaling dynamics by a series of partial differential equations (PDEs)-diffusion equations. The diffusion equation has long been developed to describe the processes with diffusive-like behavior, with or without external perturbations. A typical diffusion equation takes the following basic format:

$$\frac{du}{dt}=\overset{diffusion}{\overset{︷}{\alpha \Delta u}}+\overset{production}{\overset{︷}{\beta u}}-\overset{decay}{\overset{︷}{\gamma u+\delta }}$$

Where, u = u(x,t) is the density of the object (e.g. a type of material) studied, which is distributed in the spatiotemporal space (x,t); the first-order derivative du/dt represents the dynamic change of *u* over time; the Laplacian Δ represents the diffusion term, while the remaining two terms refer to production and decay dynamics of the object respectively; α, β, γ, δ are coefficients describing the rates of particular reactions/processes. The basic diffusion equation can be modified to adapt to particular conditions.

Each PDE depicts the diffusion, secretion and decay processes of a single molecule involved in the signaling network. For each molecule, we modified the basic diffusion equation according to its relationship with other molecules or cells revealed by previous studies, as detailed below. We solved these PDEs by finite difference method. For all PDEs, trivial Dirichlet boundary conditions were adopted. Specifically, since the diffusion occurs mainly in the center part of our computational 3D domain Ω, which is completely contained in the Bowman's capsule, the concentration of cytokines near to the domain boundary can be ignored. Therefore, suppose y is a function defined on Ω, we set y = 0 on the domain boundary ∂Ω for all the PDEs. The solutions to these PDEs will serve as important input to the cellular scale module as detailed in below section.

The hairpin-binding epidermal growth factor-like growth factor (HB-EGF) is secreted by podocytes, and exerts deleterious effect on the parietal epithelial cells (PECs) by stimulating PEC proliferation [15]. The diffusion/reaction process of HB-EGF within the glomerulus is described by the following PDE:

(1) $$\begin{array}{l} \frac{dH}{dt}=\overset{diffusion}{\overset{︷}{\alpha_{H}\Delta H}}\\ +\overset{\sec\;rection}{\overset{︷}{\chi_{Podo}\left(t\right)\lambda_{sH}\left(\text{t},\text{Podo}\right)\text{T}\left(1-H\right)}}\\ -\overset{uptake}{\overset{︷}{\chi_{PEC}\left(t\right)\lambda_{uH}H}}-\overset{decay}{\overset{︷}{{\text{d}}_{H}H}} \end{array}$$

Where, *H* and *T* refer to the normalized concentration of HB-EGF and transforming growth factor-β (TGFβ); the term *T* in the equation means that TGFβ will accelerate the HB-EGF secretion while (1-*H*) implies that the accumulation of HB-EGF will inversely suppress the HB-EGF production before it reaches the peak 1; *α_H_* and *d_H_* refer to the diffusion and decay rates of HB-EGF; *λ_sH_* and *λ_uH_* represent secretion and uptake rates of HB-EGF by podocytes and PECs respectively. We assume a constant uptake rate by PECs and a dynamic secretion rate of HB-EGF which is linearly correlated to the current podocyte health status, denoted as

$$\begin{array}{l} \lambda_{sH}\left(\text{t},\text{Podo}\right)=\lambda_{sH0}\left(Status_{Podo}\left(t\right)-1\right),\\ {\text{Status}}_{Podo}\left(t\right)=2.0\sim 2.8 \end{array}$$

In this formula, *λ*_*sH* 0_ is a basic secretion rate for normal podocytes (status = 2). *status_Podo_* (*t*) takes discrete values positively correlated with the dysfunctional state of the podocyte under investigation (with an increment of 0.2, see cellular scale of model below), directly influencing the secretion rate of HB-EGF. The term *status_Podo_* (*t*) is introduced to reflect the effect of cells on cytokine secretion, and the specific range of values is not essential since it will be assimilated by the parameter *λ*_*sH* 0_. This rule is also applicable to the other equations below.

We adopt a dynamic characteristic function χ to indicate whether a grid point is occupied by a particular type of glomerular cell at time point *t*, take podocyte as an example:

$$\chi_{Podo}\left(t\right)=\left\{ \begin{array}{l} 1,\;at\;podocyte\;grids\\ 0,\;otherwise \end{array}\right.$$

The production and diffusion of other signaling molecules can be depicted by a PDE similar to HB-EGF. The parameters and characteristic functions are set as above, with different subscripts corresponding to particular cell types or the capillary: *Endo* and *Mesg* refer to endothelial and mesangial cells respectively and *Cap* refers to the capillary network.

The vascular endothelial growth factor A (VEGF-A, denoted ‘V’) is secreted by podocytes and has a salutary effect on both endothelial cells and the podocytes [13, 14]:

(2) $$\begin{array}{l} \frac{dV}{dt}=\overset{diffusion}{\overset{︷}{\alpha_{V}\Delta V}}+\overset{\sec\;retion}{\overset{︷}{\chi_{Podo}\left(t\right)\lambda_{sV}\left(\text{t},\text{ Podo}\right)\left(1-\text{V}\right)}}\\ -\overset{uptake}{\overset{︷}{\left(\chi_{Podo}\left(t\right)\lambda_{uVp}+\chi_{Endo}\left(t\right)\lambda_{uVe}\right)\text{V}}}-\overset{decay}{\overset{︷}{{\text{d}}_{V}V}} \end{array}$$

The endothelin-1 (denoted ‘E’) is produced in podocytes upon stimulation of TGFβ (‘T’) and is harmful to endothelial and mesangial cells, as well as to podocytes [16]:

(3) $$\begin{array}{l} \frac{dE}{dt}=\overset{diffusion}{\overset{︷}{\alpha_{E}\Delta E}}+\overset{\sec\;retion}{\overset{︷}{\chi_{Podo}\left(t\right)\lambda_{sE}\left(\text{t},\text{ Podo}\right)\text{T}\left(1-\text{E}\right)}}\\ -\overset{uptake}{\overset{︷}{\left(\chi_{Podo}\left(t\right)\lambda_{uEp}+\chi_{Endo}\left(t\right)\lambda_{uEe}+\chi_{Mesg}\left(t\right)\lambda_{uEm}\right)\text{E}}}-\overset{decay}{\overset{︷}{{\text{d}}_{E}E}} \end{array}$$

The activated protein C (APC, denoted ‘A’) is produced by endothelial cells and is essential for the viability of podocytes and endothelial cells [17]:

(4) $$\begin{array}{l} \frac{dA}{dt}=\overset{diffusion}{\overset{︷}{\alpha_{A}\Delta A}}+\overset{\sec\;retion}{\overset{︷}{\chi_{Endo}\left(t\right)\lambda_{sA}\left(\text{t},\text{ Endo}\right)\left(1-A\right)}}\\ -\overset{uptake}{\overset{︷}{\left(\chi_{Podo}\left(t\right)\lambda_{uAp}+\chi_{Endo}\left(t\right)\lambda_{uAe}\right)A}}-\overset{decay}{\overset{︷}{{\text{d}}_{A}A}} \end{array}$$

where

$$\lambda_{sA}\left(\text{t},\text{Endo}\right)=\lambda_{sA0}\left(Status_{Endo}\left(t\right)-1\right),{\text{Status}}_{Endo}\left(t\right)=3.0\sim 3.8.$$

The TGFβ (denoted ‘T’) expression in mesangial cells and podocytes is induced by hyperglycemic (high glucose) stimulation but can be reduced by particular cytokine (‘C’) such as PEDF [21, 26], and can influence the health status of podocytes:

(5) $$\begin{array}{l} \frac{dT}{dt}=\overset{diffusion}{\overset{︷}{\alpha_{T}\Delta T}}\\ +\overset{\sec rection}{\overset{︷}{\left(\chi_{Podo}\left(t\right)\lambda_{sTp}\left(t,Podo\right)+\chi_{Mesg}\left(t\right)\lambda_{sTm}\left(\text{t},\text{Mesg}\right)\right)\text{G}\left(1-C\right)\left(1-T\right)}}\\ -\overset{uptake}{\overset{︷}{\chi_{Podo}\left(t\right)\lambda_{uT}T}}-\overset{decay}{\overset{︷}{{\text{d}}_{T}T}} \end{array}$$

where

$$\lambda_{sTm}\left(\text{t},\text{Mesg}\right)=\lambda_{sTm0}\left(Status_{Mesg}\left(t\right)-1\right),{\text{Status}}_{Mesg}\left(t\right)=4.0\sim 4.8.$$

The external cytokine (denoted ‘C’) and glucose (denoted ‘G’) are delivered to the glomerulus through the glomerular capillary network, and can be consumed by all four cell types with different rates:

(6) $$\frac{dC}{dt}=\overset{diffusion}{\overset{︷}{\alpha_{c}\Delta C}}+\overset{release}{\overset{︷}{\chi_{Cap}\lambda_{rC}\left(1-C\right)}}-\overset{uptake}{\overset{︷}{\left(\sum_{i}\chi_{Cell,i}\lambda_{uC,i}\right)\text{C}}}-\overset{decay}{\overset{︷}{{\text{d}}_{c}C}}$$

(7) $$\frac{dG}{dt}=\overset{diffusion}{\overset{︷}{\alpha_{G}\Delta G}}+\overset{release}{\overset{︷}{\chi_{Cap}\lambda_{rG}\left(1-G\right)}}-\overset{uptake}{\overset{︷}{\left(\sum_{i}\chi_{Cell,i}\lambda_{uG,i}\right)\text{G}}}-\overset{decay}{\overset{︷}{{\text{d}}_{G}G}}$$

Where cell type *i* takes the value of PEC, Podo, Endo or Mesg.

### Cellular scale of the model: multi-agent configuration and implementation

We treated each glomerular cell as an individual agent, and described their behaviors and activities by an agent-based model (ABM). The ABM provides a natural description of a complex system with the ability to capture emergent phenomena from the bottom up in a very flexible manner [60]. In ABM, each agent as an independent individual can communicate with its surrounding environment and make corresponding fate decisions according to a list of prescribed rules [60]. We adopted the Gaia methodology [61] to configure our agent-based model. Specifically, each cell was defined as an agent residing within the glomerulus, and different cell types were configured with different characteristics and behaviors:

(8) $$Cell\_\text{Agent }=\;<S_{t},\;{\text{E}}_{t},\;V_{t}>$$

Where, *S_t_* is a set of statuses for a particular agent at time point *t*; *E_t_* is a set of external events at time *t* which can be perceived by the agent and can exert specific effects on the agent; *V_t_* is the set of actions an agent may take at time *t*, including those spontaneous and as responses to event triggering.

The set of statuses of an agent at time *t* can be represented as:

(9) $$S_{t}=\;<p_{t},\;c_{t}>$$

Where, *p_t_* stands for the position a cell agent occupies at time *t*; *c_t_* represents the cell health status which was linearly discretized to 5 increasingly deteriorating statuses. For instance, the health status of podocytes in equation (1) was assigned from 2 to 2.8 with an increment of 0.2, in which 2 corresponds to a healthy (normal) status, and a larger value refers to a more unhealthy status.

The external events for an agent refer to the type and concentration of cytokines present in its ambient micro-environment, which can be formularized as follows:

(10) $$E_{t}=\;<H_{t},{\text{V}}_{t},{\text{E}}_{t},{\text{A}}_{t},{\text{T}}_{t},C1_{t},C2_{t},{\text{G}}_{t}>$$

Where, H_*t*_, *V_t_*, *E_t_*, *A_t_*, *T_t_* refer to dynamic local concentrations of HB-EGF, VEGF-A, endothelin-1, APC and TGFβ respectively. These five proteins are secreted and can be absorbed by particular cell types as illustrated in Figure 1B, triggering particular cell fate decision and thus playing a critical role in the cell-cell communications. *C*1_*t*_ and *C*2_*t*_ refer to external cytokines IGF1 and PEDF; *G_t_* refers to glucose concentration. In the current model, cytokines and glucose are set to be released from the glomerular capillaries and diffuse within the whole glomerulus, representing external intervention to cell biological processes.

Corresponding to the status and surrounding environment profiles, a cell agent may take a series of responses/actions at each time point *t*, which can be specifically depicted as:

(11) $$V_{t}=\;<pro,\;diff,\text{ migr, dysfun, apop}>$$

Where, *pro* and *diff* refer to cell proliferation and differentiation, *migr* refers to cell migration, and *dysfun* and *apop* refer to cell dysfunction and cell apoptosis respectively.

The implementation procedure of the multi-agent module is illustrated in Supplementary Figure S18. At each time point, the spatial distribution of the signaling molecules and imposed growth factors/glucose are determined first, by solving the molecular PDE system (1–7) as described in above section. Then, cellular behaviors and activities are updated according to their ambient signaling profiles and neighbor cells statuses. Specifically, the PEC maintains at a healthy status capable of differentiating to podocytes only if the HB-EGF is lower than a threshold level (H < H_θ_) and IGF-1 is higher than a particular level (C1 > C1_θ_) (normal +1), otherwise, the PEC proliferates and becomes hypertrophy (dysfunction) (dysfunction +1). The podocytes and endothelial cells retain healthy only if the level of endothelin-1 is low (E < E_θ_) and the levels of activated protein C and VEGF are both high (A > A_θ_, V > V_θ_) (normal +1), and become dysfunctional otherwise (dysfunction +1). The mesangial cells rely on low levels of endothelin-1 and TGFβ to maintain a normal status (E < E_θ_, T < T_θ_) (normal +1), and become dysfunctional otherwise (dysfunction +1). In our implementation, we adopted a progressive modification of cell status by a fixed increment (above section for molecular scale) instead of an abrupt change from one status to another, based on the realistic cell injury/repair processes. This progressive modification relies on a designated threshold for each molecule, and these thresholds are set as parameters in our model to facilitate later sensitivity/uncertainty analysis (Supplementary Table S2). A glomerular cell will die (apoptosis) if the dysfunction status could not be restored promptly (before the increments are exhausted). Another cellular activity is cell migration. We employed a random walk process to resolve the cell local migration as described in our earlier work [39]. Since the glomerulus has a relatively fixed structure with regular spatial distribution of glomerular cells (below section for tissue scale), the cell migration is largely confined to a prescribed region for each type of cells. This was adopted as another modeling rule.

### Tissue scale of the model: high-resolution 3D visualization and analysis

The whole simulation system was implemented in Matlab 2013a (Mathworks Inc.). We first constructed a virtual three-dimensional (3D) glomerulus which captures the major morphological structure and cellular composition/geographical distribution of the realistic glomerulus. To achieve this, a sphere representing the bowman capsule was first generated within a cubic box. Then the capillary tufts were reconstructed according to the photomicrographs of realistic anatomical structure [59]. To do so, we selected a set of grids as seeds, with spatial distributions analogous to the micrographic section, but with reduced number of nodes for simplicity. Following this step we connected these seed grids by a 3D continuous Bézier curve. Finally, we created a tubular structure along this curve to represent the real capillaries.

Four types of glomerular cells were initially seeded into the generated glomerulus according to their real location within the glomerulus[62]: parietal epithelial cells (‘PEC’) were seeded on the internal surface of the bowman capsule; endothelial cells (‘Endo’) and podocytes (‘Podo’) were set to line the surface and outside of the capillaries respectively; mesangial cells (‘Mesg’) were seeded in the interstitial space among podocytes and endothelial cells. Initial cell numbers were determined from literatures [63]: Podo = 900, Endo = Mesg = 1300, and PECs were estimated as PEC = 600.

For a better visualization, the membrane of bowman capsule and surface of capillaries were configured transparent with different colors and transparencies. Cells were represented by closed or open symbols with different shapes and colors. At each time point of simulation, cell numbers of different types will be counted, and the concentration of each signaling proteins will be checked to track the cellular and molecular dynamics within the glomerulus, respectively. And these measurements will be combined to an overall evaluation of the efficiency of podocyte regeneration.

### Parameter determination and estimation

The proposed systems model involved two sets of parameters, pertaining to the molecular and cellular modules respectively. The molecule module parameters (Supplementary Table S1) referred to the diffusion, secretion and uptake/degradation rate of signaling proteins involved, including HB-EGF, VEGF-A, Endothelin-1, APC and TGFβ. It also referred to physics properties of the external cytokines and glucose release and diffusion processes. These parameters were present as coefficients in the partial differential equations (PDEs) used to describe the mentioned molecular processes. The cellular module parameters (Supplementary Table S2) involved a batch of thresholding and scaling factors of specific signaling proteins for particular cell fate decision. This group of parameters was set to configure the probability thresholds in the agent-based model for cellular activities such as differentiation and status maintenance.

We estimated these parameters by iteratively calibrating them within a prescribed range until the following two outcomes were achieved: first, the cell numbers could maintain a dynamic equilibrium under normal condition and second, under diabetic high glucose condition, the cell numbers were reduced by a particular percentage comparable to previous clinical observations [18]. To facilitate efficient search of the desired parameters, we fixed the parameters related to secretion rates of all molecules (Supplementary Table S1). We also assumed similar uptake rate of one particular protein by different cell types for simplicity. Since we only considered the trend over time and relative levels of different proteins, all the molecule concentrations were made dimensionless and normalized to the range between 0 and 1 in equations (1–7). And thus the production term (secretion/release rate) could be prescribed and fixed without emphasizing on their specific values. Corresponding to the dimensionless property of the variables, all the parameters, including the coefficients in equations (1–7) and thresholds in Supplementary Figure S18 can be also deemed nondimensional, representing relative rates instead of real measurements [64].

### Parameter sensitivity and model uncertainty analysis

The parameter sensitivity analysis was conducted to assess the sensitivity of the model output to the involved parameters. We checked the total and healthy cell number changes upon perturbations on specific parameters. Specifically, at one time we perturbed one parameter by 10% from its baseline value, and calculated the relative changes in the cell numbers at the last simulation time point. This procedure was conducted only on parameters which were not fixed and distinct from others (Supplementary Table S1). When one parameter was perturbed, the remaining parameters were kept unchanged. And for each case, we calculated cell number variations for all four glomerular cell types.

The model uncertainty analysis was performed to evaluate the robustness of the model subject to randomness configurations. The agent-based model contained certain randomness prescribed to account for the cellular responses to the dynamic change of surrounding physiological conditions, resulting in potential oscillation in the model output. To measure this uncertainty, we repeated the simulations for five times for each of the treatment conditions with baseline parameter settings, and calculated the coefficients of variance at each time point *CV_t_* as the ratio of the standard deviation to the average cell number of the repeats at that time point: *CV_t_* = *S_t_* / M_*t*_.

## SUPPLEMENTARY MATERIALS TABLE FIGURES


