# Supplementary material for: Intraglomerular crosstalk elaborately regulates podocyte injury and repair in diabetic patients: insights from a 3D multiscale modeling study
Source: Oncotarget. 2016 Sep 24;7(45):73130–46. doi: 10.18632/oncotarget.12233 (PMC5341968; doi:10.18632/oncotarget.12233)
Supplement: Supplementary file 1 [file oncotarget-07-73130-s001.pdf]

Intraglomerular crosstalk elaborately regulates podocyte injury and repair in diabetic patients: insights from a 3D multiscale modeling study

Supplementary Materials

SUPPLEMENTARY RESULTS

Alternative diffusion model by integrating convection process

We tested an alternative model for signaling molecule diffusion process by integrating the convection process. Since the convection only takes place inside the capillaries, we imposed an indicator function χ_{Cap} to restrict the domain of convection to the capillary regions. Therefore, the basic diffusion model in the main text

$$\frac{du}{dt} = \overbrace{\alpha\Delta u}^{diffusion} + \overbrace{\beta u}^{production} - \overbrace{(\gamma u + \delta)}^{decay}$$

is modified to

$$\frac{du}{dt} = \overbrace{\alpha\Delta u}^{diffusion} - \overbrace{\delta\chi_{Cap}\nabla\cdot u}^{convection} + \overbrace{\beta u}^{production} - \overbrace{(\gamma u + \delta)}^{decay}$$

Where δ refers to convection coefficient and χ_{Cap} is defined as in equations (6) and (7) in the text, and the other parameters in the equations are defined as those in the original model.

With this modification, the molecular diffusion model (1)-(5) can be accordingly modified as the following (1a–5a). Since the cytokine and glucose are delivered through the arterioles (the capillaries), the convection loss can be excluded from the equations since we only consider the net remainder of the released molecules after offsetting the convection loss.

$$\frac{dH}{dt} = \overbrace{\alpha_H\Delta H}^{diffusion} - \overbrace{\nabla\cdot(\delta_H\chi_{Cap}\,\mathrm{H})}^{convection} + \overbrace{\chi_{Podo}(t)\lambda_{sH}(\mathrm{t},\mathrm{Podo})\,\mathrm{T}(1-H)}^{secretion} - \overbrace{\chi_{PEC}(t)\lambda_{uH}H}^{uptake} - \overbrace{\mathrm{d}_H H}^{decay} \quad (1a)$$

$$\frac{dV}{dt} = \overbrace{\alpha_V\Delta V}^{diffusion} - \overbrace{\nabla\cdot(\delta_V\chi_{Cap}\,\mathrm{V})}^{convection} + \overbrace{\chi_{Podo}(t)\lambda_{sV}(\mathrm{t},\mathrm{Podo})(1-\mathrm{V})}^{secretion} - \overbrace{(\chi_{Podo}(t)\lambda_{uVp} + \chi_{Endo}(t)\lambda_{uVe})\,\mathrm{V}}^{uptake} - \overbrace{\mathrm{d}_V V}^{decay} \quad (2a)$$

$$\frac{dE}{dt} = \overbrace{\alpha_E\Delta E}^{diffusion} - \overbrace{\nabla\cdot(\delta_E\chi_{Cap}\,\mathrm{E})}^{convection} + \overbrace{\chi_{Podo}(t)\lambda_{sE}(\mathrm{t},\mathrm{Podo})\,\mathrm{T}(1-E)}^{secretion} - \overbrace{(\chi_{Podo}(t)\lambda_{uEp} + \chi_{Endo}(t)\lambda_{uEe} + \chi_{Mesg}(t)\lambda_{uEm})\,\mathrm{E}}^{uptake} - \overbrace{\mathrm{d}_E E}^{decay} \quad (3a)$$

$$\frac{dA}{dt} = \overbrace{\alpha_A\Delta A}^{diffusion} - \overbrace{\nabla\cdot(\delta_A\chi_{Cap}\,\mathrm{A})}^{convection} + \overbrace{\chi_{Endo}(t)\lambda_{sA}(\mathrm{t},\mathrm{Endo})(1-A)}^{secretion} - \overbrace{(\chi_{Podo}(t)\lambda_{uAp} + \chi_{Endo}(t)\lambda_{uAe})A}^{uptake} - \overbrace{\mathrm{d}_A A}^{decay} \quad (4a)$$

$$\frac{dT}{dt} = \overbrace{\alpha_T\Delta T}^{diffusion} - \overbrace{\nabla\cdot(\delta_T\chi_{Cap}\,\mathrm{T})}^{convection} + \overbrace{(\chi_{Podo}(t)\lambda_{sTp}(t,Podo) + \chi_{Mesg}(t)\lambda_{sTm}(\mathrm{t},\mathrm{Mesg}))\,\mathrm{G}(1-C)(1-T)}^{secretion} - \overbrace{\chi_{Podo}(t)\lambda_{uT}T}^{uptake} - \overbrace{\mathrm{d}_T T}^{decay} \quad (5a)$$

We conducted a series of simulations on the podocyte regeneration process based on the alternative model. The parameters in the original model were kept unchanged. The additional parameters (δ_*) were varied by an order of magnitude 1~4, i.e., δ = 0.001, 0.01, 0.1, 1. The cell status distribution evolution, healthy cell number dynamics, and dynamic change of critical cytokines under normal (physiologic) conditions are illustrated in Supplementary Figures S19–S21, respectively. The model output for high glucose condition is shown in Supplementary Figures S22–S24 correspondingly.

From these figures it can be seen that, the major model output only varied little from that of the baseline model, i.e., the scenario of δ = 0. Under the physiologic condition, when the coefficient δ takes 0.1, which is comparable to the other parameters in the order of magnitude (Supplementary Table S1), the relative variation in cell number comparing to the baseline model is bounded by 3% across all simulation time points (Supplementary Figure S20). This tendency remains true

in the high glucose scenario (Supplementary Figure S23). The variation of scenario δ = 0.1 in cell status frequency distribution and cytokine dynamics relative to baseline profiles can also be seen from the following figures. Actually, in all simulations, the results of scenarios δ = 0.01 and δ = 0.001 only differs negligibly from those of the baseline scenario. These results indicate that the convection term does not significantly impact the instant and relative distribution of the cytokines within the glomerulus, and hence rarely influence the dynamic change of cell numbers.

On the other hand, when the convection coefficient δ takes extremely large value, for example, δ = 1 as shown in the simulation, the model output at the molecular level deviates substantially from the baseline situation. This tendency is especially obvious for VEGF-A and APC (Supplementary Figures S21 and S24), leading to significant variation in number of healthy glomerular cells, especially of endothelial cells (Supplementary Figures S20 and S23). It should be noted that we leveraged physiologic and hyperglycemic conditions to calibrate our model for further treatment analysis. Therefore, the molecular and cellular dynamic profiles without treatment is enforced to corroborate the realistic dynamics, as elucidated in the main text. The optimization process will guide the system to determine the best parameters including the convection coefficient δ, restricting them within an appropriate range. If δ takes large values (e.g. δ ≥ 1), then the production rate λ can be revalued accordingly to offset the concentration loss due to increased convection, which can be seen from the original model (1–5) and the alternative model

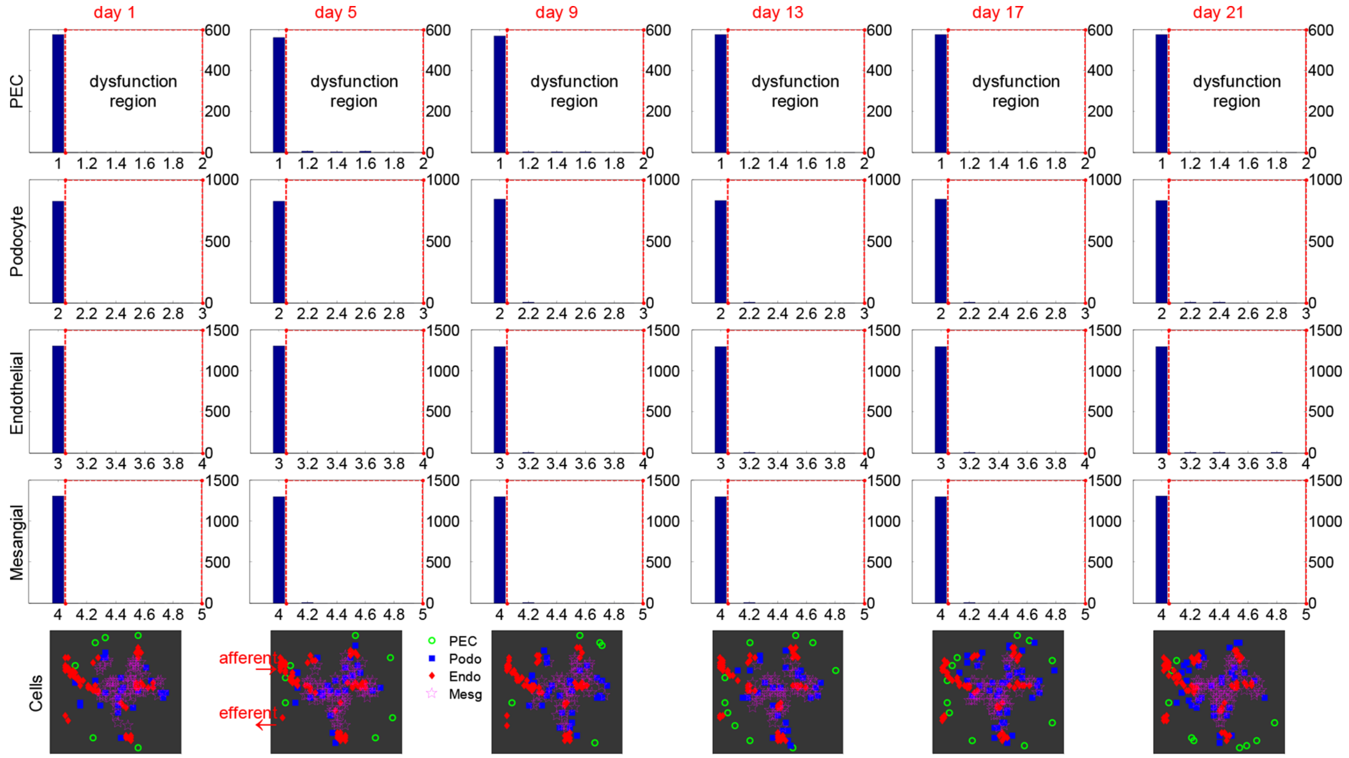

Supplementary Figure S1: Glomerular cell status frequency and geographic distribution in 2D section view over simulation time under physiologic condition. The first four rows refer to the cell status frequency of parietal epithelial cell (PEC), podocyte, endothelial and mesangial cell, respectively. All cells manifested normal status during the simulation time, since few cells were distributed in the dysfunction region. The last row illustrates the cell geographic distribution at section $y = 0$. Afferent and efferent arterioles are denoted as red arrows in the second subfigure of the last row.

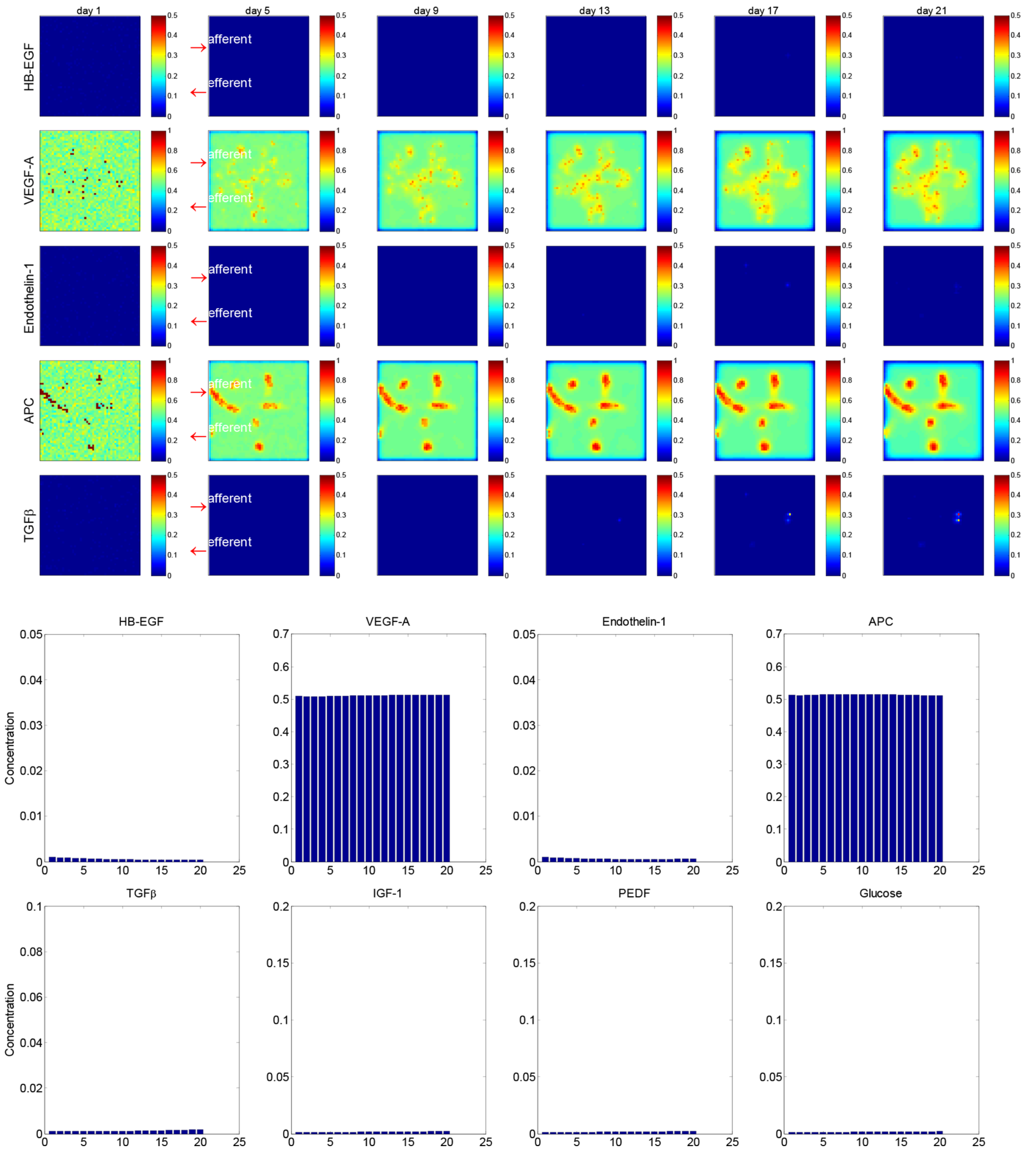

Supplementary Figure S2: Dynamic change of critical cytokines within glomerulus under normal condition. The upper panel shows the glomerulus-wide distribution and concentration of the cytokines in 2D section view. Cytokine names are denoted on the left side of each row. The lower panel is a quantified representation of the molecule concentration dynamics corresponding to the upper panel. External IGF-1, PEDF, and Glucose remained negligible concentration during the whole simulation, indicating the normal (physiologic) condition for this simulation.

(1a–5a).

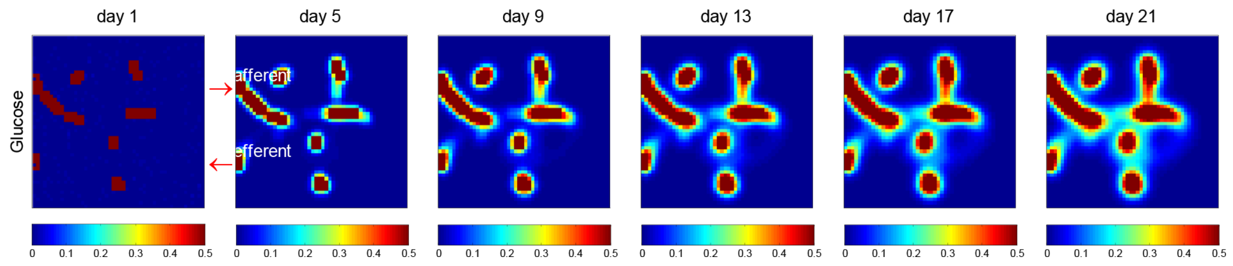

Supplementary Figure S3: A 2D section view of glucose diffusion within the glomerulus over a period of 21 simulation days. Glucose extravasates from the capillaries and diffuses into the glomerulus. Afferent and efferent arterioles are indicated by red arrows in the second subfigure.

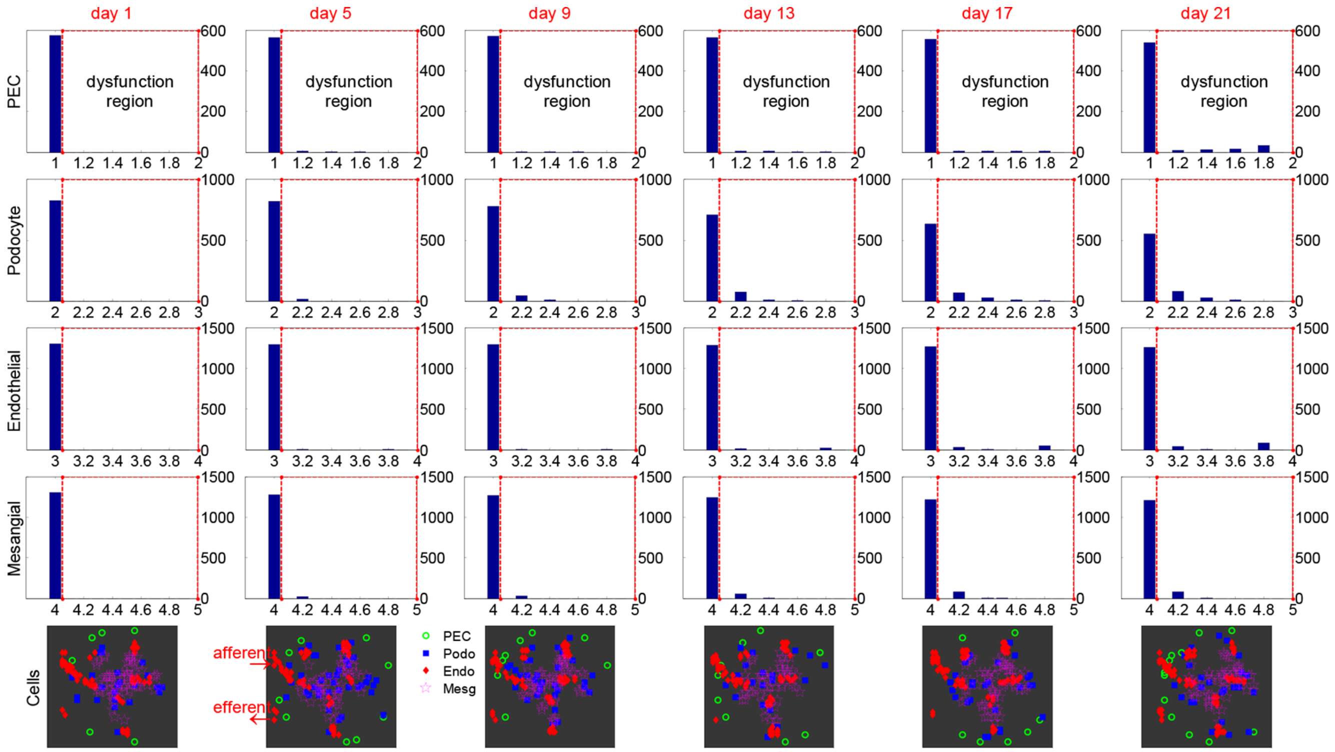

Supplementary Figure S4: Glomerular cell status frequency and geographic distribution in 2D section view over simulation time under hyperglycemic condition. The first four rows refer to the cell status frequency of parietal epithelial cell (PEC), podocyte, endothelial and mesangial cell, respectively. All cells began to manifest unhealthy status during the simulation time, since a significant proportion of cells were distributed in the dysfunction region. The last row illustrates the cell geographic distribution at section y=0. Afferent and efferent arterioles are denoted as red arrows in the second subfigure of the last row.

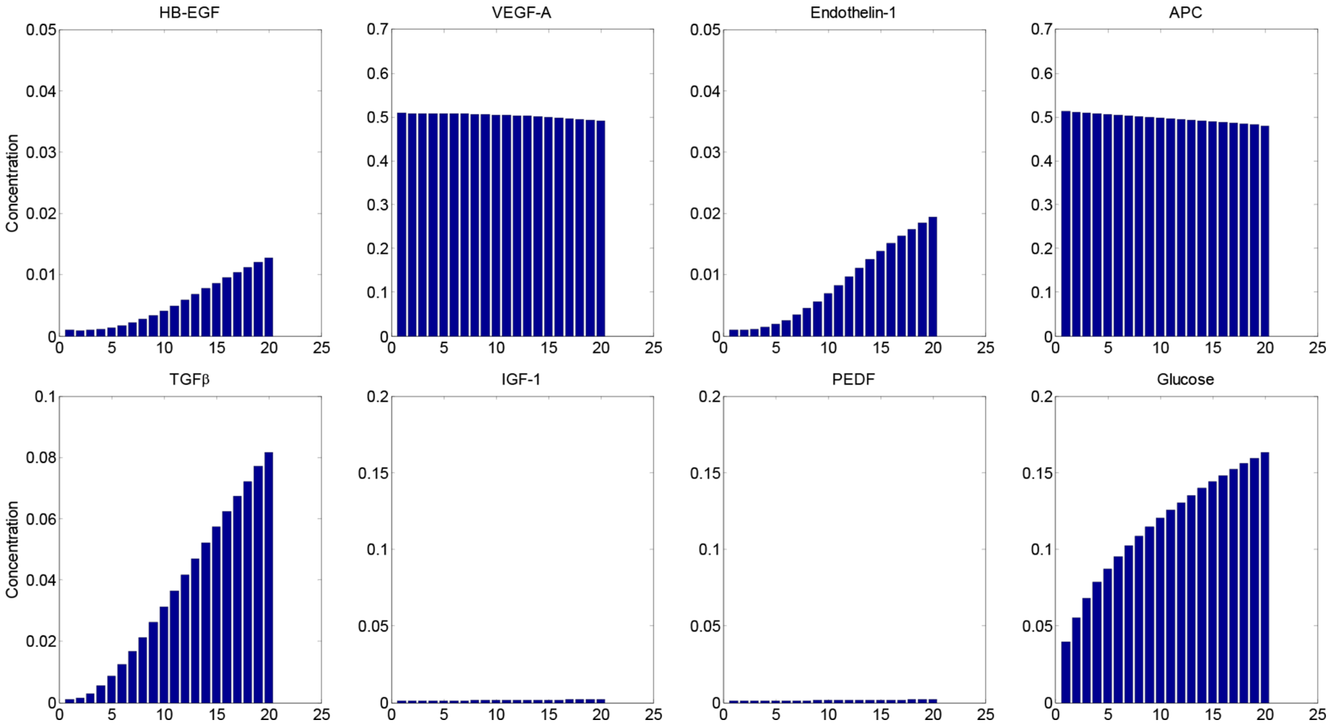

Supplementary Figure S5: Dynamic change of critical cytokines within glomerulus under high glucose condition. Shown is a quantified representation of the molecule concentration dynamics corresponding to Figure 4. External IGF-1 and PEDF remained negligible concentration, while the glucose was continuously increased during the simulation period, indicating the hyperglycemic condition for this simulation.

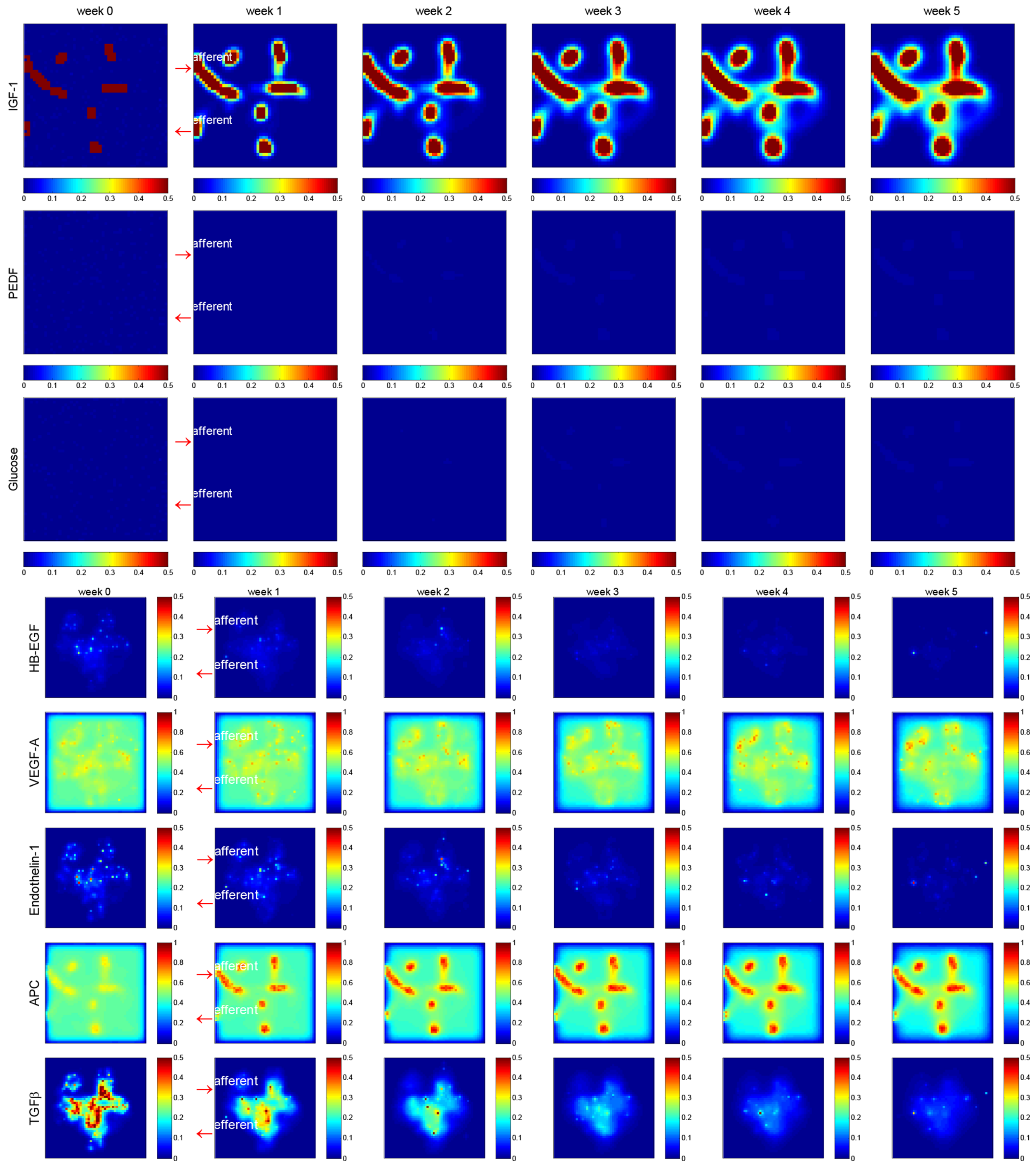

Supplementary Figure S6: Molecular concentration and distribution dynamics under IGF-1 treatment at normal glucose level. Rows 1–3 show the treatment condition: IGF-1 treatment alone at normal glucose level; Rows 4–8 illustrate concentration and distribution dynamics of important signaling molecules over simulated treatment time (week 0–5): HB-EGF, Endothelin-1 and TGFβ were attenuated significantly, while VEGF-A and APC were maintained at high levels. Afferent and efferent arterioles were indicated by red arrows in the second subfigure in each row.

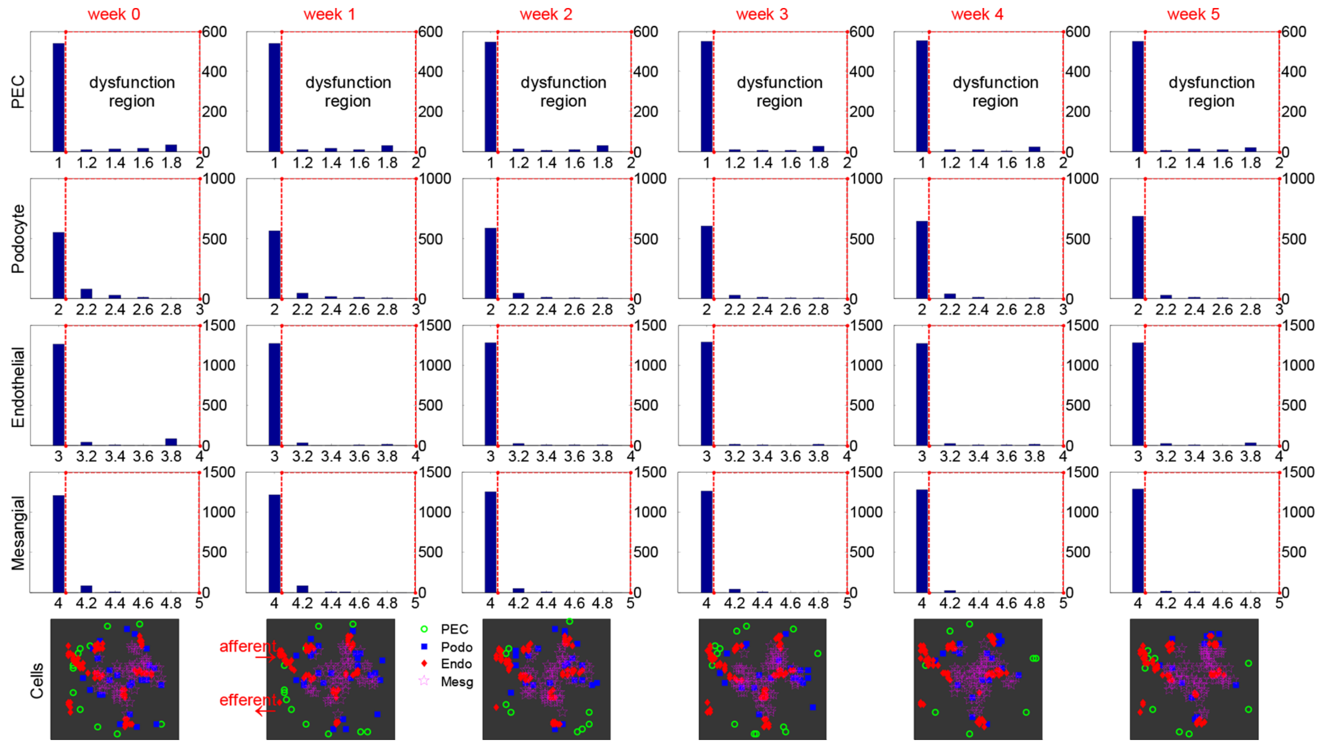

Supplementary Figure S7: Glomerular cell status frequency and geographic distribution in 2D section view over simulation time under IGF-1 treatment. The first four rows refer to the cell status frequency of parietal epithelial cell (PEC), podocyte, endothelial and mesangial cell, respectively. All cells began to return to normal status during the simulation time, since number of cells in the dysfunction region began to diminish over time. The last row illustrates the cell geographic distribution at section $y = 0$. Afferent and efferent arterioles are denoted as red arrows in the second subfigure of the last row.

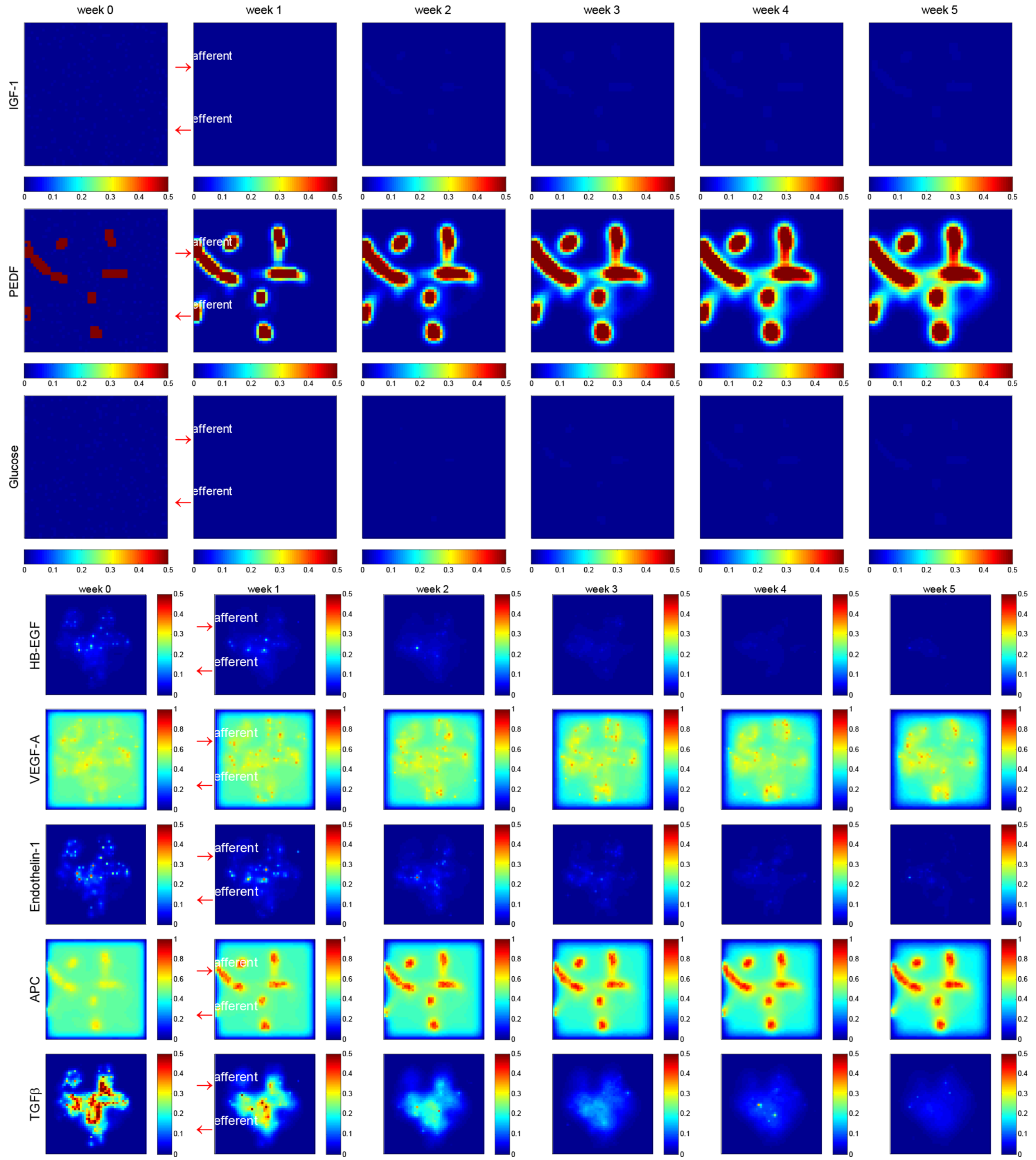

Supplementary Figure S8: Molecular concentration and distribution dynamics under PEDF treatment at normal glucose level. Rows 1–3 show the treatment condition: PEDF treatment alone at normal glucose level; Rows 4–8 illustrate concentration and distribution dynamics of important signaling molecules over simulated treatment time (week 0–5): HB-EGF, Endothelin-1 and TGFβ were attenuated significantly, while VEGF-A and APC were maintained at high levels. Afferent and efferent arterioles were indicated by red arrows in the second subfigure in each row.

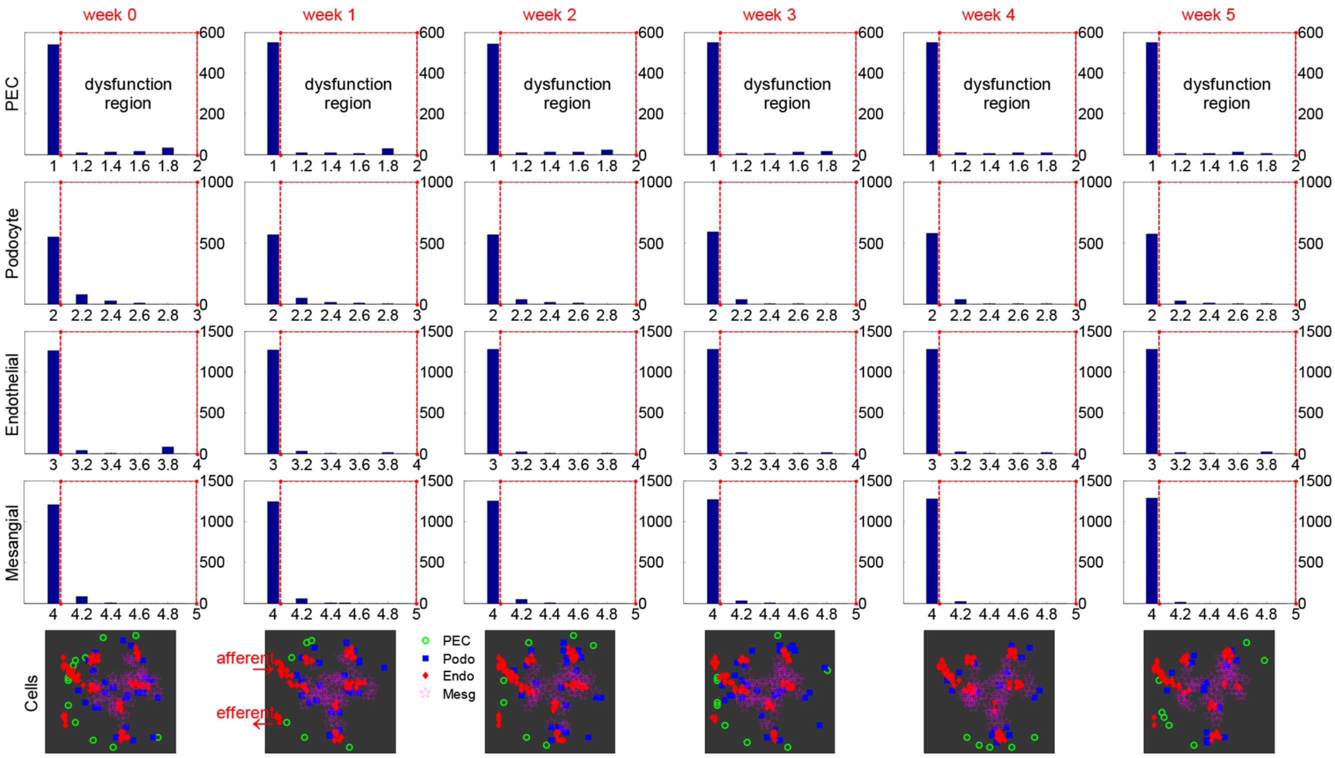

Supplementary Figure S9: Glomerular cell status frequency and geographic distribution in 2D section view over simulation time under PEDF treatment. The first four rows refer to the cell status frequency of parietal epithelial cell (PEC), podocyte, endothelial and mesangial cell, respectively. All cells began to return to normal status during the simulation time, since number of cells in the dysfunction region began to diminish over time. The last row illustrates the cell geographic distribution at section $y = 0$. Afferent and efferent arterioles are denoted as red arrows in the second subfigure of the last row.

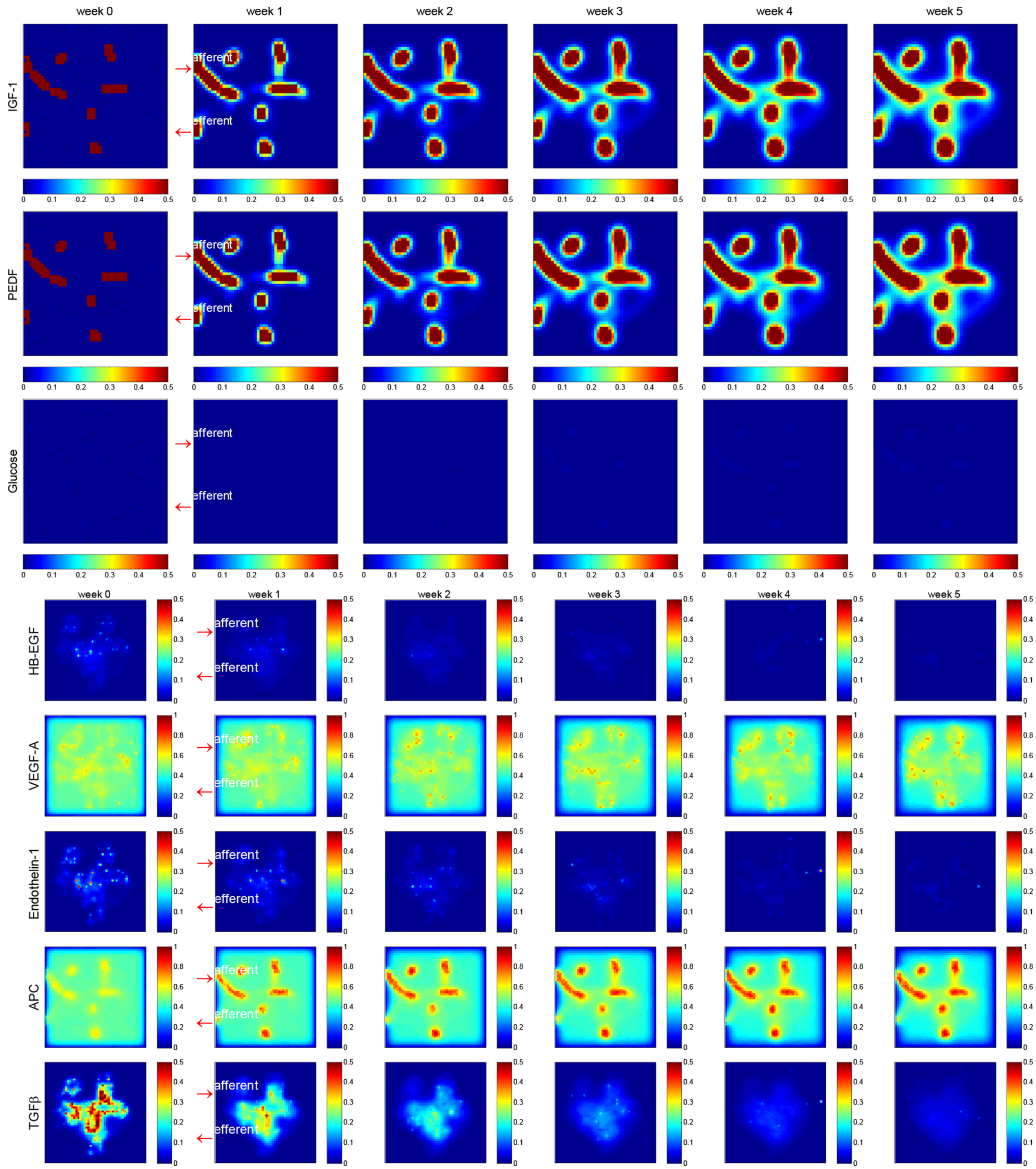

Supplementary Figure S10: Molecular concentration and distribution dynamics under IGF-1 and PEDF combined treatment at normal glucose level. Rows 1–3 show the treatment condition: IGF-1 and PEDF combined treatment at normal glucose level; Rows 4–8 illustrate concentration and distribution dynamics of important signaling molecules over simulated treatment time (week 0–5): HB-EGF, Endothelin-1 and TGFβ were attenuated significantly, while VEGF-A and APC were maintained at high levels. Afferent and efferent arterioles were indicated by red arrows in the second subfigure in each row.

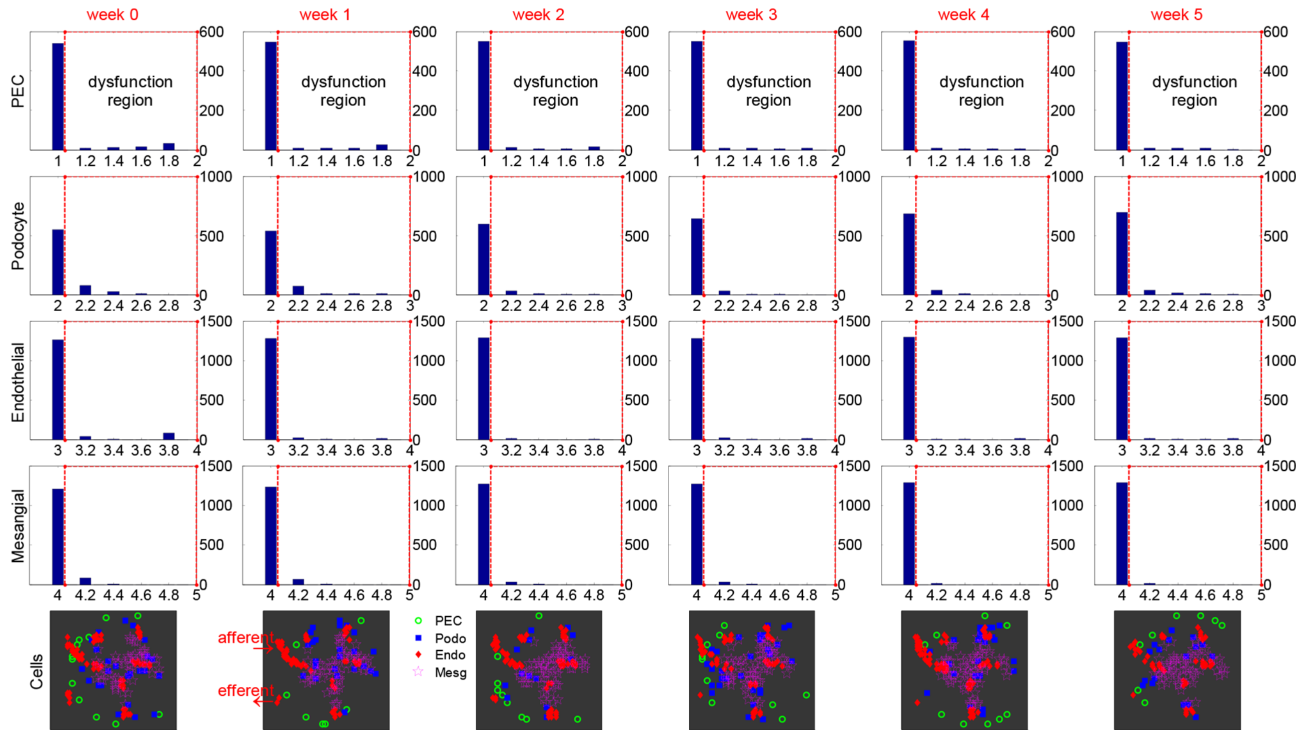

Supplementary Figure S11: Glomerular cell status frequency and geographic distribution in 2D section view over simulation time under IGF-1 and PEDF combined treatment. The first four rows refer to the cell status frequency of parietal epithelial cell (PEC), podocyte, endothelial and mesangial cell, respectively. All cells returned to normal status rapidly during the simulation time, as shown by the prompt decline of cells in the dysfunction region. The last row illustrates the cell geographic distribution at section $y = 0$. Afferent and efferent arterioles are denoted as red arrows in the second subfigure of the last row.

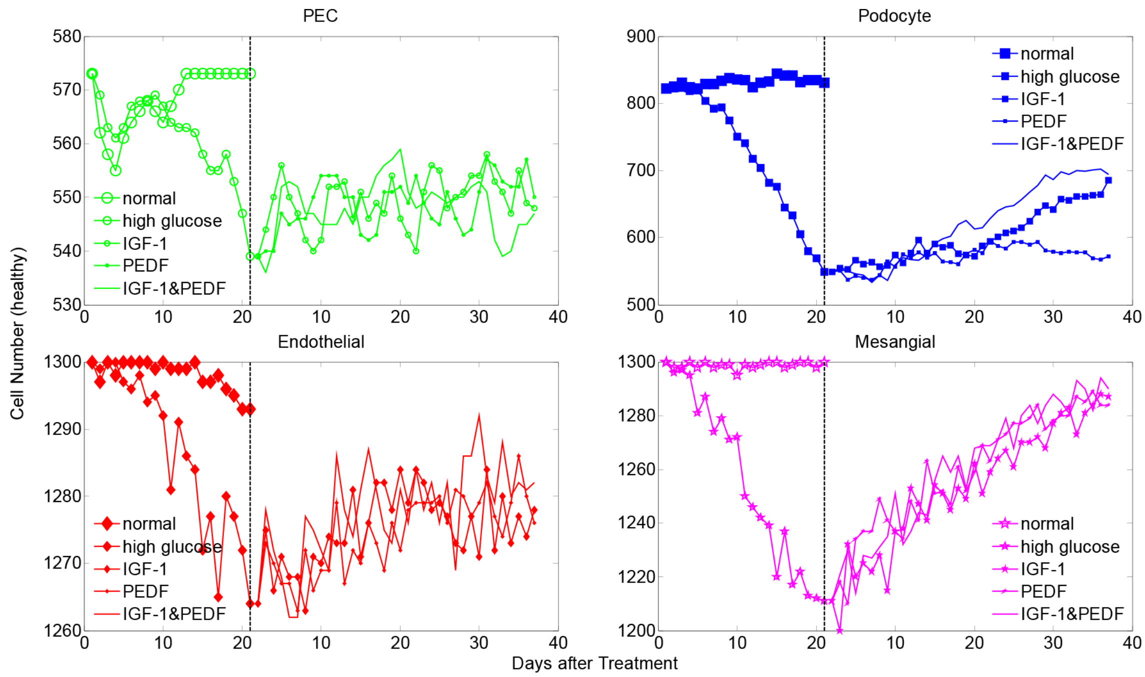

Supplementary Figure S12: Healthy cell number change under single and dual cytokine treatments. Shown are healthy cell numbers of each glomerular cell type under denoted treatments after high-glucose induced injury. The cell number dynamics under normal (i.e. physiologic) condition is plotted for comparison.

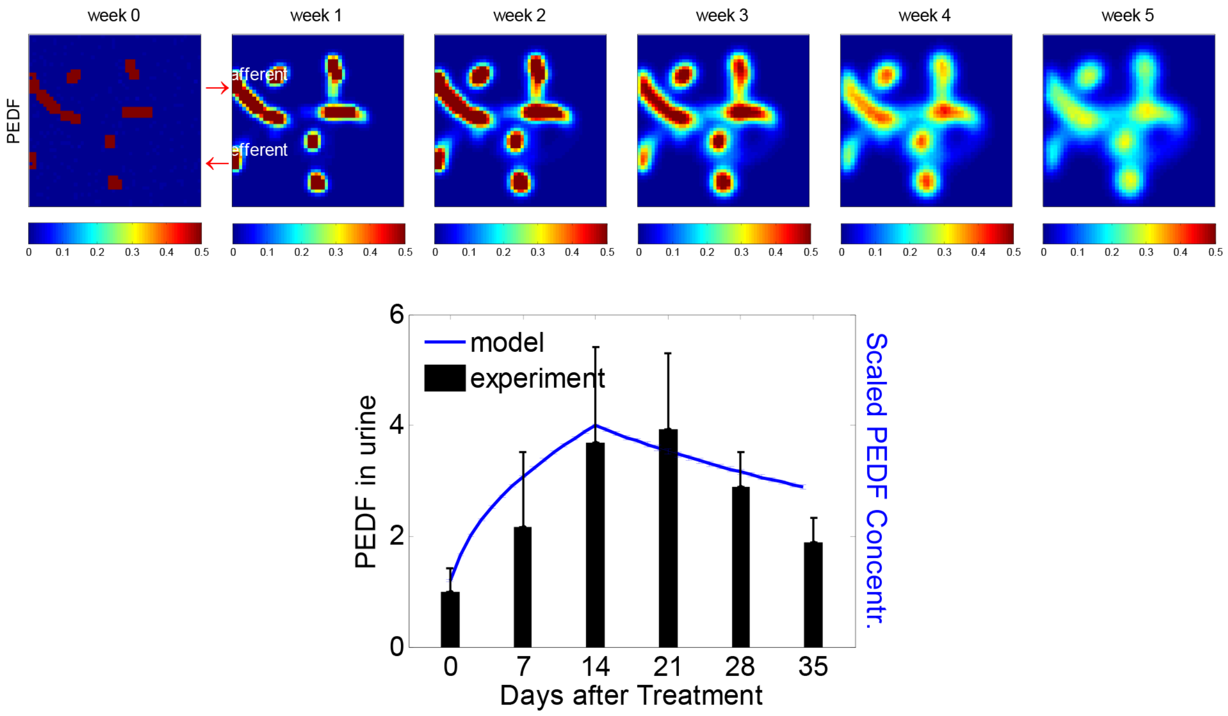

Supplementary Figure S13: An alternative simulated PEDF treatment schedule comparable to our published *in vivo* experiments, to facilitate the comparison of treatment outcomes between simulation and experiments. Upper panel: 2D section view of simulated PEDF distribution within glomerulus. Lower panel: quantification of simulated PEDF dynamics (blue curve) and experimental observation of urine PEDF excretion after PEDF treatment in diabetic rats (black bars, see ref. 21).

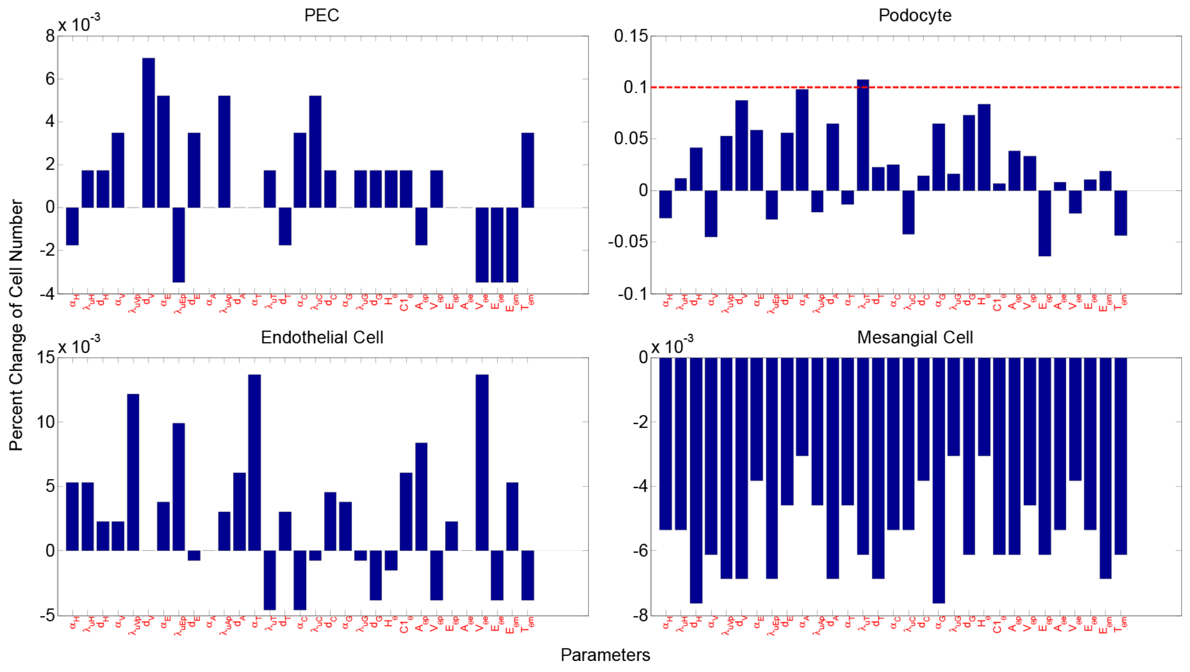

Supplementary Figure S14: Parameter sensitivity analysis results. Shown are percent cell number change of total glomerular cells at week 5 upon 10% increase in parameters.

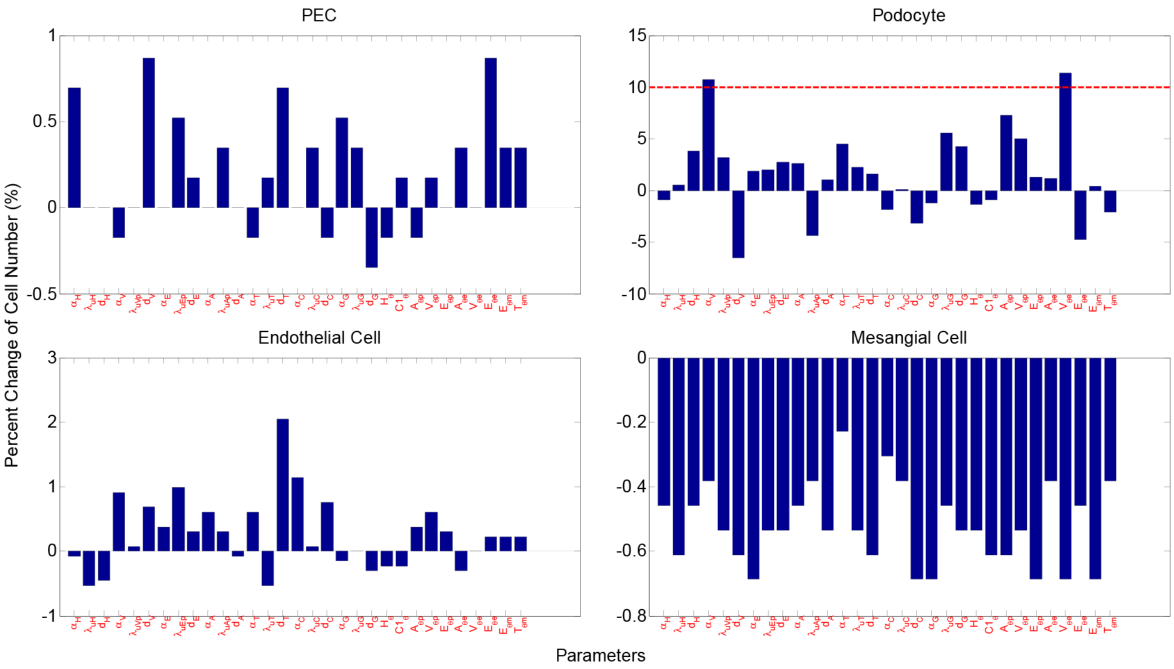

Supplementary Figure S15: Parameter sensitivity analysis results. Shown are percent cell number change of total glomerular cells at week 5 upon 10% decrease in parameters.

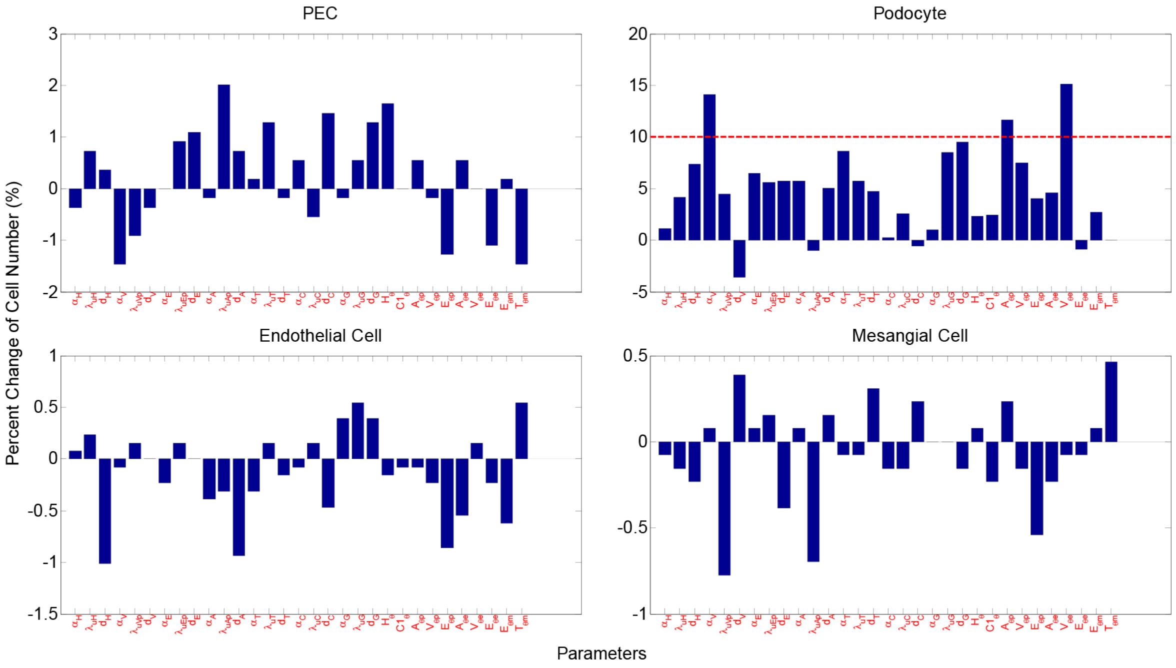

Supplementary Figure S16: Parameter sensitivity analysis results. Shown are percent cell number change of healthy glomerular cells at week 5 upon 10% decrease in parameters.

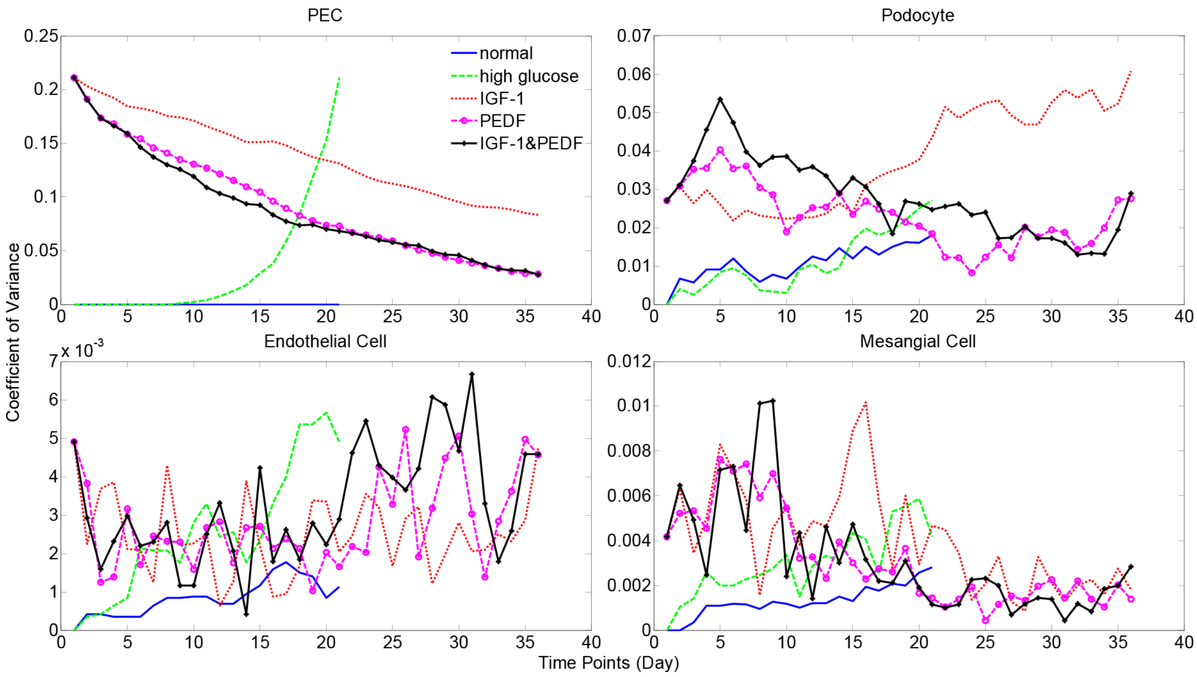

Supplementary Figure S17: Model uncertainty analysis results for total cell numbers. Simulations were repeated five times with identical parameter settings. Coefficient of variance (CV) was calculated as the ratio of standard deviation to mean cell number at each time point. A small CV (<1) indicates stability of the model.

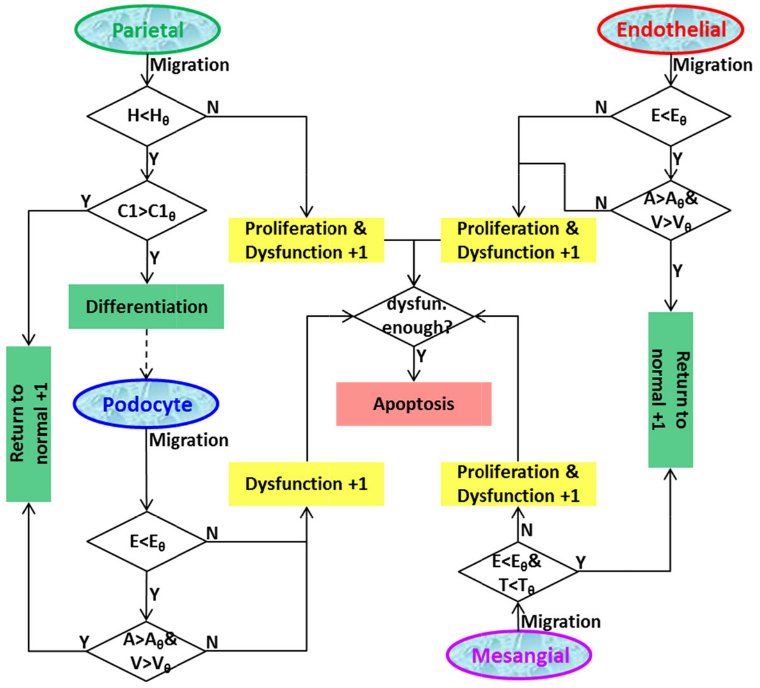

Supplementary Figure S18: Implementation of the multi-agent model. Colored ellipsoids refer to the four resident glomerular cell types; Diamonds represent the conditions for cell fate decision; Rectangles correspond to the cell fate decisions (green: normal; yellow: abnormal). Molecule abbreviation is defined as: H: HB-EGF; V: VEGF-A; E: Endothelin-1; A: activated protein C (APC); T: TGFβ; C1: Cytokine 1 (refers to IGF-1). The subscript θ indicates prescribed threshold value for that particular molecule. +1 means that the cell health status (normal or dysfunction) changes by one increment. More details about the configuration of the agents and implementation of the cellular scale module are provided in the Materials and Methods section.

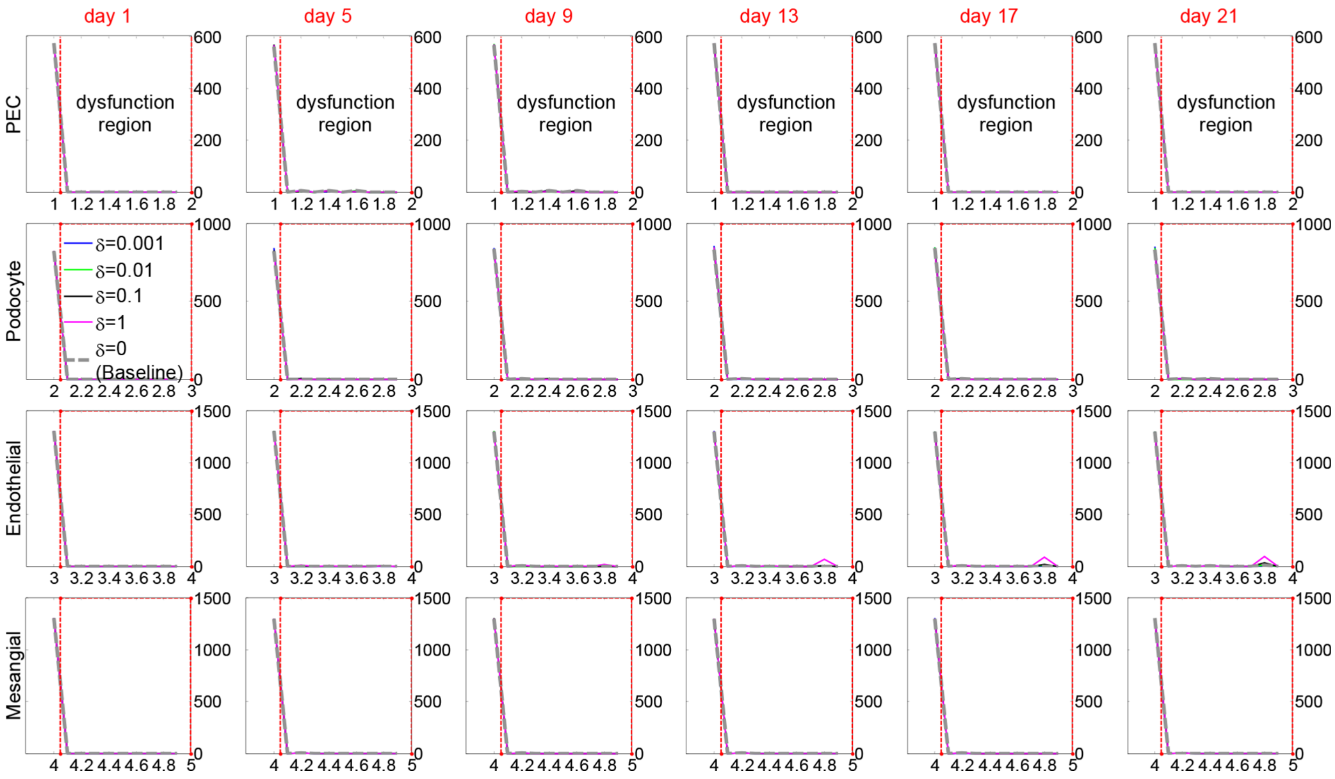

Supplementary Figure S19: Glomerular cell status frequency distribution over simulation time under normal (physiologic) condition with distinct convection rates. $\delta = 0$ refers to the baseline model without consideration of the convection effect.

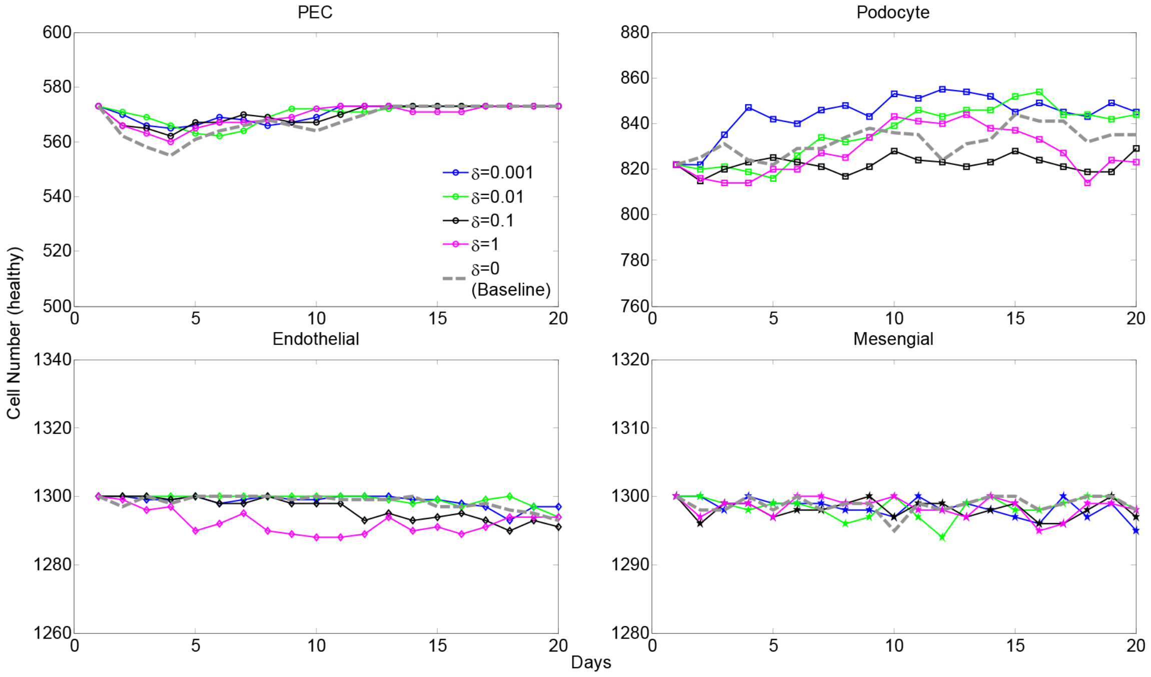

Supplementary Figure S20: Healthy cell number dynamics under physiologic condition with distinct convection rates. $\delta = 0$ refers to the baseline model without consideration of the convection effect.

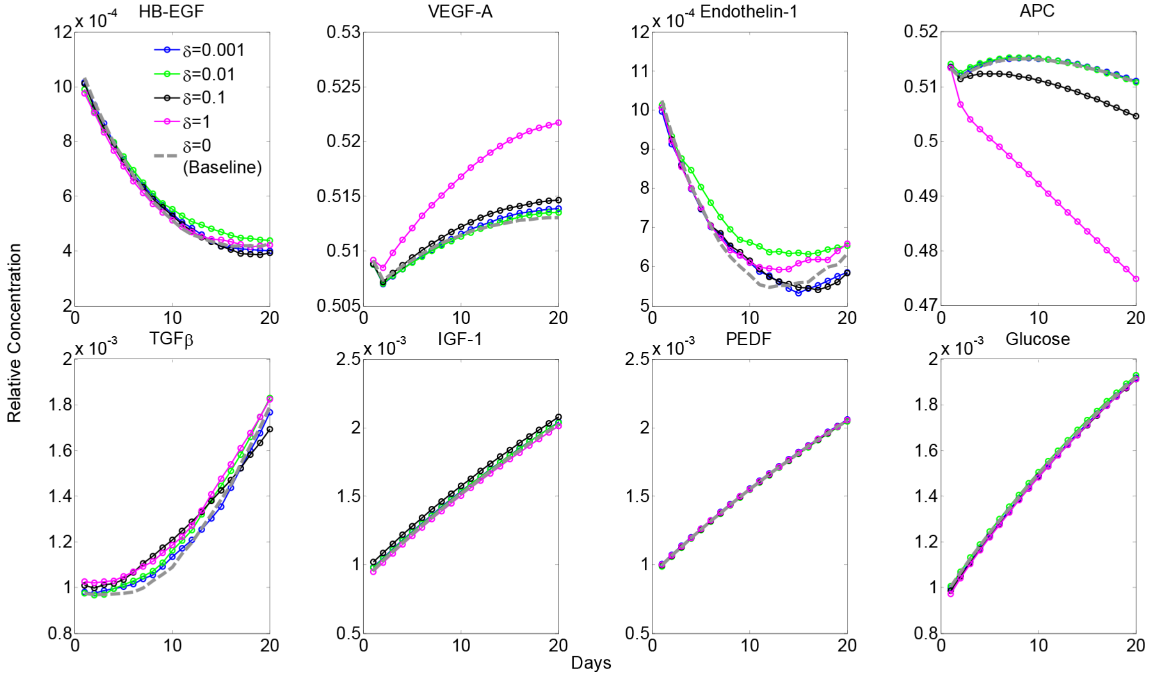

Supplementary Figure S21: Dynamic change of critical cytokines under physiologic condition with distinct convection rates. δ = 0 refers to the baseline model without consideration of the convection effect. External IGF-1, PEDF, and Glucose remained negligible concentration during the whole simulation, indicating the normal (physiologic) condition for this simulation.

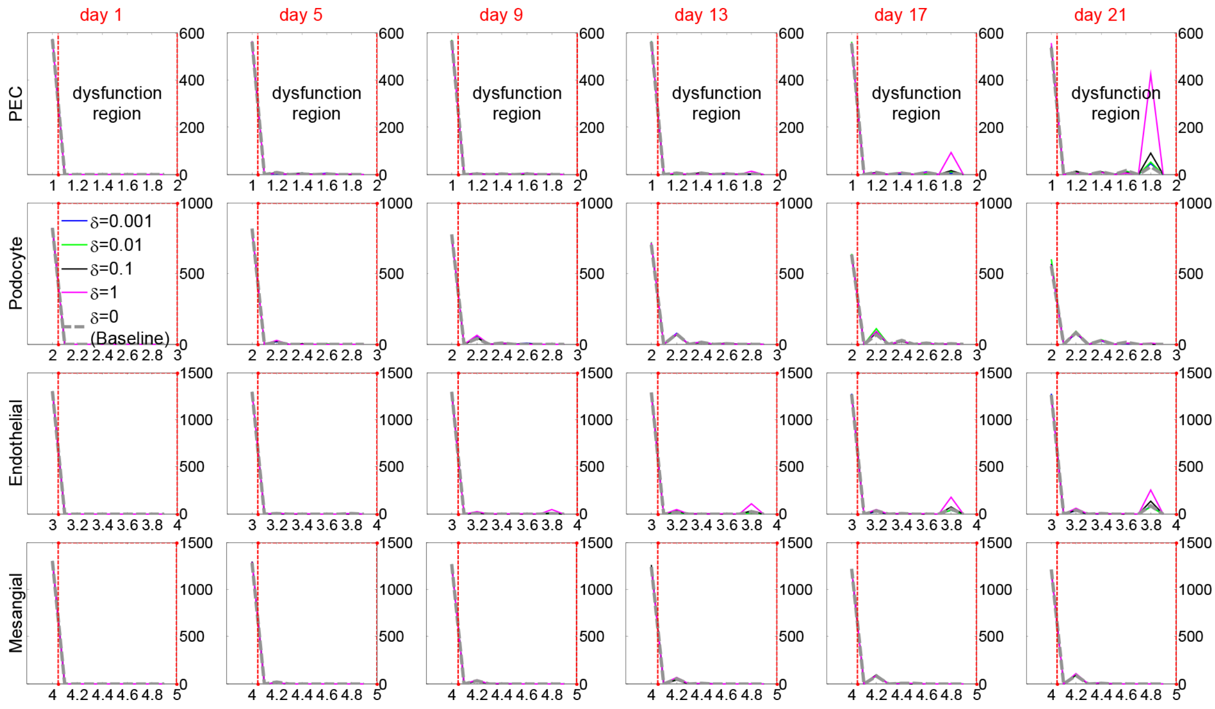

Supplementary Figure S22: Glomerular cell status frequency distribution over simulation time under high glucose condition with distinct convection rates. δ=0 refers to the baseline model without consideration of the convection effect.

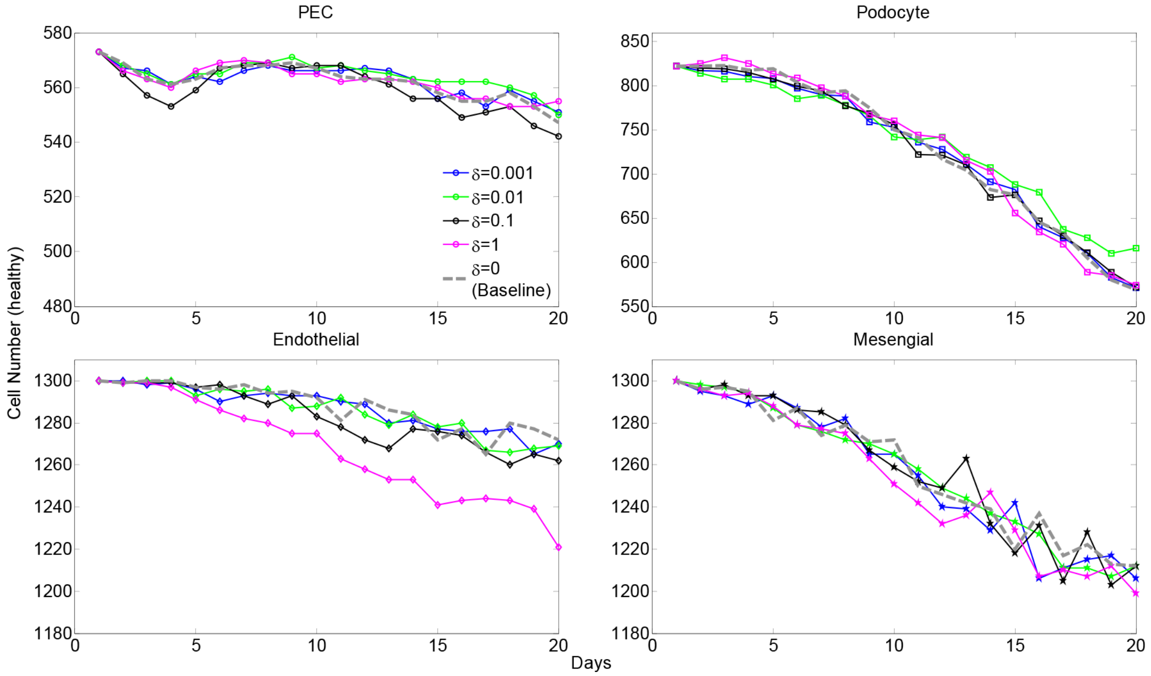

Supplementary Figure S23: Healthy cell number dynamics under high glucose condition with distinct convection rates. $\delta = 0$ refers to the baseline model without consideration of the convection effect.

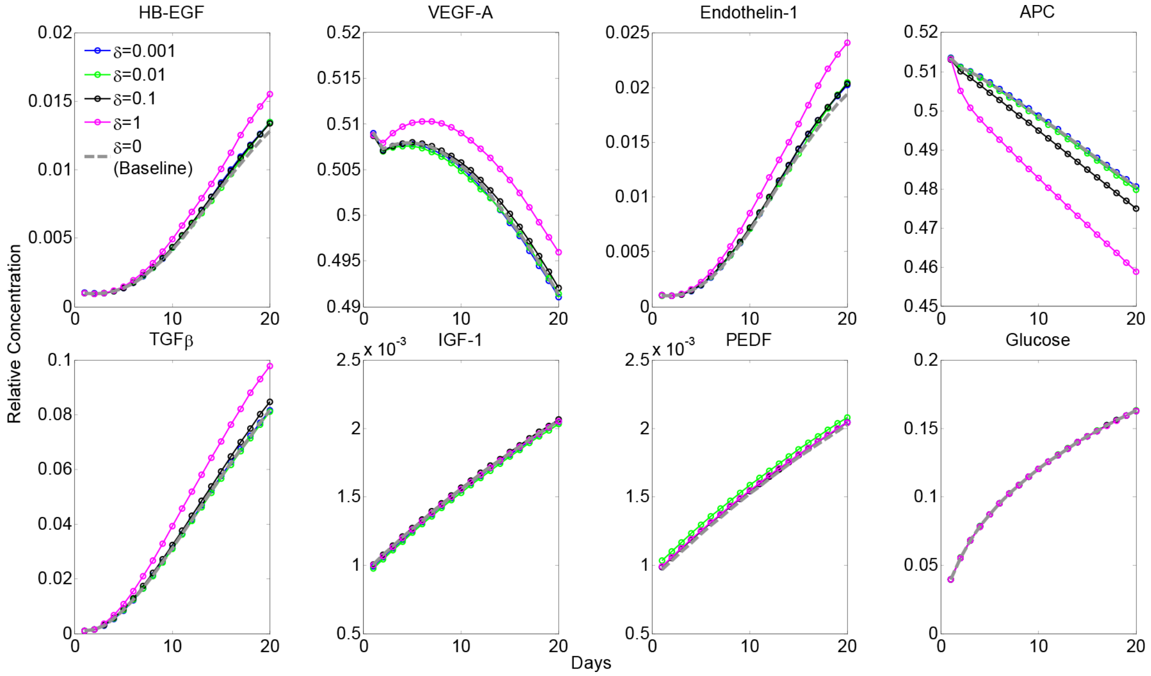

Supplementary Figure S24: Dynamic change of critical cytokines under high glucose condition with distinct convection rates. $\delta = 0$ refers to the baseline model without consideration of the convection effect. External IGF-1 and PEDF remained negligible concentration, while the glucose was continuously increased during the simulation period, indicating the hyperglycemic condition for this simulation.

Supplementary Table S1: Nondimensional parameters used in the molecular scale module

ID	Symbol	Description	Value	Range
**1**	α_H	Diffusion coefficient of HB-EGF	0.5	[0:0.125:0.875]*
**2**	λ_{sHo}	Secretion rate of HB-EGF by podocyte	0.5	Fixed
**3**	λ_{uH}	Uptake rate of HB-EGF by PEC	0.1	[0:0.05:0.2]
**4**	d_H	Degradation rate of HB-EGF	0.1	[0:0.05:0.2]
**5**	α_V	Diffusion coefficient of VEGF-A	0.125	[0:0.125:0.875]
**6**	λ_{sVo}	Secretion rate of VEGF-A by podocyte	0.5	Fixed
**7**	λ_{uVp}	Uptake rate of VEGF-A by podocyte	0.01	[0:0.005:0.02]
**8**	λ_{uVe}	Uptake rate of VEGF-A by endothelial cell	0.01 = λ_{uVp}	N/A
**9**	d_V	Degradation rate of VEGF-A	0.005	[0:0.005:0.02]
**10**	α_E	Diffusion coefficient of Endothelin-1	0.25	[0:0.125:0.875]
**11**	λ_{sEo}	Secretion rate of Endothelin-1 by podocyte	1	Fixed
**12**	λ_{uEp}	Uptake rate of Endothelin-1 by podocyte	0.1	[0:0.05:0.2]
**13**	λ_{uEp}	Uptake rate of Endothelin-1 by endothelial cell	0.1 = λ_{uEp}	N/A
**14**	λ_{uEm}	Uptake rate of Endotheilin-1 by mesangial cell	0.1 = λ_{uEp}	N/A
**15**	d_E	Degradation rate of Endothelin-1	0.1	[0:0.05:0.2]
**16**	α_A	Diffusion coefficient of APC	0.125	[0:0.125:0.875]
**17**	λ_{sAo}	Secretion rate of APC by endothelial cell	0.5	Fixed
**18**	λ_{uAp}	Uptake rate of APC by podocyte	0.01	[0:0.005:0.02]
**19**	λ_{uAp}	Uptake rate of APC by endothelial cell	0.01 = λ_{uAp}	N/A
**20**	d_A	Degradation rate of APC	0.005	[0:0.005:0.2]
**21**	α_T	Diffusion coefficient of TGFβ	0.125	[0:0.125:0.875]
**22**	λ_{sTpo}	Secretion rate of TGFβ by podocyte	0.5	Fixed
**23**	λ_{sTm0}	Secretion rate of TGFβ by mesangial cell	0.5	Fixed
**24**	λ_{uT}	Uptake rate of TGFβ by podocyte	0.1	[0:0.05:0.2]
**25**	d_T	Degradation rate of TGFβ	0.05	[0:0.05:0.2]
**26**	α_C	Diffusion coefficient of cytokine	0.1	[0:0.05:0.5]
**27**	λ_{rC}	Release rate of cytokine from capillary	0.5	Fixed
**28**	λ_{uC}	Uptake rate of cytokine by glomerular cells	0.05	[0:0.005:0.1]
**29**	d_C	Degradation rate of cytokine	0.005	[0:0.005:0.1]
**30**	α_G	Diffusion coefficient of glucose	0.15	[0:0.05:0.5]
**31**	λ_{rG}	Release rate of glucose from capillary	0.5	Fixed
**32**	λ_{uG}	Uptake rate of glucose by glomerular cell	0.05	[0:0.005:0.1]
**33**	d_G	Degradation rate of glucose	0.01	[0:0.005:0.1]

*[a:b:c] stands for parameters were searched from a to c with an increment of b.

These parameters appear in the equations (1–7) serving as the diffusion/production/decay rates of the signaling proteins in the molecular module. Parameters in each partial differential equation (PDE) formed an individual parameter group which was coded by a particular background color.

Supplementary Table S2: Nondimensional parameters used in the cellular scale module

ID	Symbol	Description	Value	Range
**34**	H_θ	Threshold of HB-EGF for PEC damage	5×10^{-4}	[4.5×10^{-4} 5.5×10^{-4}]
**35**	$C1_\theta$	Scaling factor of IGF-1 for podocyte differentiation	3.6	[3.24 3.96]
**36**	$A_{\theta p}$	Scaling factor of APC for podocyte recovery	2.25	[2.025 2.475]
**37**	$V_{\theta p}$	Scaling factor of VEGF-A for podocyte recovery	2.25	[2.025 2.475]
**38**	$E_{\theta p}$	Scaling factor of Endothelin-1 for podocyte damage	1	[0.9 1.1]
**39**	$A_{\theta e}$	Scaling factor of APC for endothelial cell recovery	5	[4.5 5.5]
**40**	$V_{\theta e}$	Scaling factor of VEGF-A for endothelial cell recovery	5	[4.5 5.5]
**41**	$E_{\theta e}$	Scaling factor of Endothelin-1 for endothelial cell damage	1	[0.9 1.1]
**42**	$E_{\theta m}$	Scaling factor of Endothelin-1 for mesangial cell damage	1	[0.9 1.1]
**43**	$T_{\theta m}$	Scaling factor of TGFβ for mesangial cell damage	0.1	[0.09 0.11]

These parameters appear in Figure S18 specifying the switch conditions for cell fate decisions in the cellular module. Background colors discriminate between parameter groups involved in different cell types.
